# Annotation of bat IG H/L/K loci and analysis of the characteristics of bat BCR-CDR3 repertoires

**DOI:** 10.3389/fimmu.2026.1827051

**Published:** 2026-05-20

**Authors:** QinLu Liu, Long Ma, Jun Li, LongYu Liu, Hao Zhou, ZeGang Liu, HuiFang Wang, XinSheng Yao

**Affiliations:** Department of Immunology, Zunyi Medical University, Guizhou Provincial Research Center for Applied Immunomolecular Engineering, Guizhou Provincial Innovation Base for Graduate Education in Immunology, Guizhou Provincial Key Discipline of Immunology, Zunyi, China

**Keywords:** *Antrozous pallidus*, BCR CDR3 repertoire, high-throughput sequencing, IG locus annotation, *Rhinolophus ferrumequinum*

## Abstract

Bats represent the second largest order of mammals and are the only flying mammals, exhibiting remarkable biological characteristics such as viral tolerance, longevity, and low tumor incidence. Although bats serve as reservoirs for numerous zoonotic viruses, they remain asymptomatic without overt infection. However, the mechanisms and effects of B-cell adaptive immune responses in bats are largely unknown. In mammals, BCR V(D)J gene recombination and the formation of the CDR3 repertoire are key mechanisms underlying B-cell diversity, specificity, and memory responses. In this study, we performed, for the first time, a chromosome-level annotation of the IGK locus (17 KV; 4 KJ;1 LC) and IGL locus (74 LV; 6 LJ-LC gene clusters) in *Rhinolophus ferrumequinum*, as well as the IGH locus (81 HV; 16 HD; 6 HJ;HC of IgM, IgG, IgE, IgA) and IGL locus (56 LV; 9 LJ-LC gene clusters) in *Antrozous pallidus.* Comparative evolutionary analyses of each IG locus were also conducted. Using the annotated IG H/K/L constant region gene sequences, we employed 5′ Rapid Amplification of cDNA Ends (5′RACE) to construct BCR CDR3 repertoires and perform high-throughput sequencing (HTS). The annotated bat IG H/K/L genes were then used to build a MixCR tool for bat BCR CDR3 HTS analysis. In the spleen and intestinal tissues of bats from *Hipposideros armige* and *Rhinolophus pearsonii/pusillus*, we observed high diversity and differential preferential usage of V and J genes in the BCR CDR3 repertoires. Compared with human and mouse BCR CDR3 repertoires, bat CDR3 repertoires exhibited both similarities (e.g., high-frequency motifs, tyrosine enrichment) and distinct differences (e.g., N/P nucleotide Additions/Deletions patterns, amino acid distribution in the CDR3 region). This study provides chromosome-level annotations of IG loci in bats from different families and preliminarily reveals the fundamental characteristics of the bat BCR-CDR3 repertoire, along with key differences compared to human and mouse. Our findings offer comparative base-data, novel insights, tools, and strategies for future in-depth investigations into B-cell adaptive immune responses in bats.

## 1 Introduction

Bats are the only flying mammals, comprising approximately 1,400 species across about 20 families (1, 2), and are classified into two suborders: *Megachiroptera* and *Microchiroptera* (3, 4). Bats exhibit long lifespans, low tumor incidence, and carry numerous highly pathogenic viruses without frequently showing clinical symptoms (5). Their immune responses differ markedly from those of other mammals, attracting significant research attention. To date, a variety of severe zoonotic viruses have been detected in bats, including Marburg virus, Nipah virus, rabies virus, Ebola virus, and Severe Acute Respiratory Syndrome(SARS) coronavirus (6–10). Early hypotheses suggested that flight-induced elevation of body temperature suppresses viral replication in bats (11). Recent studies have demonstrated that bats inhibit inflammation by suppressing the transcriptional initiation of the NOD-like receptor family, pyrin domain containing 3 (NLRP3), leading to reduced function of the interferon (IFN)-inducible protein absent in melanoma 2 (AIM2), decreased activity of cysteine-requiring aspartate protease 1 (caspase-1), and impaired interleukin-1β (IL-1β) cleavage (12–14). Bats also utilize various immune molecules, including IFNs, to suppress viruses (15–18).

Since the identification of mammalian-like B cells in *Pteropus giganteus* by electron microscopy in 1991 (19), multiple lines of evidence suggest that bat B cells may play a dominant role in viral tolerance or response. For example, stimulation with lipopolysaccharide (LPS) in *Pteropus alecto* increases B cell numbers in the spleen and blood (20). Upon ex vivo stimulation with staphylococcal enterotoxin B (SEB) or LPS of peripheral blood mononuclear cells and splenocytes from adult and juvenile *Rousettus aegyptiacus*, B cell proliferation was detected in both age groups (21). Bats infected with rabies virus produce neutralizing antibodies (22). Infections with other viruses have also shown elevated IgG antibody levels and the presence of virus-neutralizing antibodies *in vivo* (23–25). However, research on bat B cell composition, response effects, and mechanisms lags significantly, particularly the annotation of immunoglobulin (IG) light chain loci at the chromosome level and the analysis of the BCR CDR3 repertoire, which remain unexplored. Early work by Baker et al. annotated 23 IGHV sequences and 5 IGH segments in *Pteropus alecto*, and 74 IGHV sequences in *Pteropus vampyrus* via mRNA cloning (26). Larson et al. annotated the IGH locus of the Egyptian fruit bat using the IMGT database, Ig-BLAST, and recombination signal sequence (RSS) analysis. Using the Geneious Prime tool and mapping germline genes from IMGT-recorded species, we previously performed the first chromosome-level annotations of the IGH locus in *Rhinolophus ferrumequinum*, *Phyllostomus discolor*, and *Pipistrellus pipistrellus* (27), as well as the T cell receptor (TR) loci (28, 29). In this study, we screened high-quality bat genome sequencing data from public databases such as NCBI, and based on the available chromosome-level assemblies of *Rhinolophus ferrumequinum* and *Antrozous pallidus*, the main objectives are: (1) Perform the first annotation of IGK and IGL loci in bats from different families and conduct comparative evolutionary analyses. (2) Construct BCR CDR3 libraries and perform high-throughput sequencing (HTS) to characterize the BCR-CDR3 repertoires (heavy and light chains) in different bat species and tissues, and to preliminarily compare the major similarities and differences between bat, human, and mouse BCR CDR3 repertoires.

## 2 Materials and methods

### 2.1 Annotation workflow for the IG H/L locus of *Antrozous pallidus* and the IG L/K locus of *Rhinolophus ferrumequinum*

(1) Download the chromosome-level genome assembly sequences of *Antrozous pallidus* (NCBI accession: GCA_027563665.1) and *Rhinolophus ferrumequinum* (NCBI accession: GCA_00415265.2) from the NCBI database.

(2) Following the IMGT annotation pipeline for IG loci, use the conserved constant (C) region genes to locate the IG loci. Employ the BLAST tool to retrieve homologous sequences in bat genomes using mammalian IGHC, IGLC, and IGKC sequences provided by IMGT, thereby determining the chromosomal positions of the bat IGH, IGL, and IGK loci. Based on previously identified bat light chain boundary genes, BLAST them onto the bat IGL/K chromosomal loci, and integrate germline gene annotation results to determine whether boundary genes exist in the bat IG L/K loci.

(3) Use Geneious Prime software with sequence alignment functionality to map the downloaded mammalian heavy chain and light chain gene sequences onto the bat chromosomal loci containing IG H/L/K, thereby obtaining putative genes. Place the confirmed locus regions into the Ig-BLAST tool to screen for potentially unannotated genes.

(4) Use the Rss-database website to identify germline genes that may have been missed due to unannotated recombination signal sequences (RSS).

(5) Criteria for gene identification. V gene: contains a complete open reading frame with a length greater than 271 bp. D gene: short nucleotide sequence with high GC content (<30 bp), located between V and J gene clusters, flanked by a 12-RSS and a 23-RSS respectively. J gene: contains a conserved motif at the end of the coding region, approximately 50–60 bp in length. C gene: composed of multiple exons, including constant region domains.

### 2.2 Characterization and nomenclature of germline genes at the IG H/L/K loci of *Antrozous pallidus* and *Rhinolophus ferrumequinum*

(1) According to IMGT rules, analyze the germline gene characteristics of *Rhinolophus ferrumequinum* and *Antrozous pallidus*. Using the IMGT/V-Quest tool, divide the nucleotide sequences of heavy chain (IGHV, IGHD, IGHJ) and light chain (IGLV, IGLJ; IGKV, IGKJ) genes from *Antrozous pallidus* and *Rhinolophus ferrumequinum* into functional regions (FR1-4; CDR1-3). Based on IMGT functional classification criteria, define three categories of genes: functional genes (F), open reading frames (ORF), and pseudogenes (P).

(2) Use Geneious Prime software to analyze the nucleotide and amino acid similarity of V, D, and J genes within the IG H/L/K loci of *Antrozous pallidus* and *Rhinolophus ferrumequinum*, and perform visual analysis of the amino acid backbone structure and conserved amino acid positions.

(3) Use the alignment function of Geneious Prime software to group *Antrozous pallidus* and *Rhinolophus ferrumequinum* IGHV/IGLV/IGKV genes with nucleotide similarity ≥75% into the same family. For V genes with nucleotide similarity between 65% and 75%, determine whether they belong to the same family based on their position in the phylogenetic tree. Select genes from species closely related to bats to construct a phylogenetic tree, and based on the tree topology, make a final determination of family assignment. Name the families according to the names of gene families from species confirmed to belong to the same family.

(4) Use the EMBOSS Dotmatcher tool to analyze the duplication patterns of V, D, J, and C genes within the bat IGH/IGL/IGK loci, and compare with gene families recorded in IMGT from other species.

(5) Using MEGA11 and TimeTree software, select species recorded in IMGT (Artiodactyla, Primates, Rodentia, Carnivora, Salmoniformes, platypus, chicken, rabbit, horse, etc.) together with bats *(Chiroptera)* to analyze gene evolution. Use MEGA11 software to construct phylogenetic trees of V and C genes from species closely related to bats (human, mouse, dog, cattle, pig) as well as other species recorded in IMGT, and analyze the evolutionary characteristics of bat genes compared with other species.

(6) Use the Weblogo 3 tool to analyze the sequence characteristics of the 12RSS/23RSS located upstream or downstream of V and J genes in the IG H/L/K loci of *Antrozous pallidus* and *Rhinolophus ferrumequinum*, and compare with those of humans and mice.

### 2.3 Construction of the MixCR (v4.0) analysis tool for the bat IG H/L/K CDR3 repertoire

Referring to the previously established reference libraries for three bat IGH genes (27), and based on the high gene homology between *Antrozous pallidus* and *Rhinolophus ferrumequinum*, we used the annotated IGL/K genes to construct, for the first time, bat IGL and IGK reference gene libraries using MixCR (v4.0). The resulting bat gene reference libraries can be directly read by the MixCR tool for high-throughput sequencing (HTS) analysis of the bat BCR-CDR3 repertoire.

### 2.4 Bat sample preparation and BCR CDR3 HTS

Bat Muscle, spleen, and intestinal tissues were collected by trained personnel (Zunyi Medical University Animal Ethics Committee, Approval No: Lun Shen [2018] No. 2-176). Under strict biosafety precautions, three Greater Horseshoe Bats (*Hipposideros armiger*), 6 Pearson’s horseshoe bats (*Rhinolophus pearsonii*), and 1 Pygmy horseshoe bat (*Rhinolophus pusillus*). In accordance with bat sample collection protocols, the captured bats were euthanized via decapitation, and DNA was extracted from the muscle tissue for species identification of the Cytb gene (**Supplementary Table 1**). RNA was extracted from bat spleen and intestinal samples, and a BCR CDR3 Repertoires were constructed using the 5’RACE method. Based on our annotated IG K/L constant region (C) sequences and all bat C-region sequences currently shared on NCBI (**Supplementary Figure 1**: 29 species for IGLC; 21 species for IGKC), we designed 5’RACE primers capable of covering the C regions of multiple bat species (with some bases substituted by X). The 5’RACE primers used in this experiment are located at the 5’ end of the IgK/L constant region (C). The 5’RACE repertoire construction primer for IGKC is: 5’GGTXGGAAGATGAAGXXGGATGG; the 5’RACE repertoire construction primer for IGLC is: ACCGAGGGXGCXGACTTGGGCTG; the 5’RACE repertoire construction primer for IGHC is: IgA (TTGAGGCTCAGCGGGAAGAC); IgE(AAGACGGAG GGGCTTGTCCT); IgG(GAACACTGACGGAGCCGTTTTAG); IgM(CTCACAAGACACGA GTGGGAAGAG); The 5’ RACE primers for IGH CDR3 are described in our previously published article (27). After successful construction of the CDR3 Repertoires, perform high-throughput screening (HTS) on the BCR CDR3 repertoires, generate raw sequencing data, and upload it to a shared database. Analyze the composition and characteristics of bat BCR CDR3 sequences using the MiXCR analysis tool, which was developed based on a bat reference genes.

### 2.5 Comparative analysis of BCR CDR3 repertoires among bats, humans, and mice

Peripheral blood samples from 4 healthy volunteers (male, aged 19–25 years) were subjected to HTS of the IGH BCR CDR3 repertoire (**Supplementary Table 2**, Ethics Committee of Zunyi Medical University, Approval No [2021].1-022). Bone marrow samples from 4 female Balb/c mice (3 months old) were subjected to HTS of the IGH BCR CDR3 repertoire (**Supplementary Table 3**, Animal Ethics Committee of Zunyi Medical University, Approval No [2021].2-040). Additionally, public databases were queried to download IGL and IGK BCR CDR3 repertoire data from human peripheral blood and mouse spleen and bone marrow constructed using the 5′RACE method (**Supplementary Table 2**; **Supplementary Table 3**. Note: Some of the human and mouse control samples were derived from the same source as those used in our previously published study (27)), for comparative analysis of IGH/IGL/IGK CDR3 repertoires among bats, humans, and mice. Using immunarch (0.9.1), VDJtools (1.2.1), EXCEL, and other software, the IGH/IGL/IGK CDR3 repertoires of bats, humans, and mice were analyzed for V and J gene usage and V-J pairing, CDR3 length distribution and amino acid (AA) usage, Non-templated(N)/Palindromic(P) nucleotide Additions/Deletions patterns in the CDR3 region, CDR3 diversity, clonality, overlap (using inverse Simpson and Shannon indices to calculate and evaluate CDR3 repertoire diversity), as well as tracking and comparative analysis of public CDR3 sequences/motifs.

### 2.6 Statistical analysis and graphing

Graphs were generated using R language (version 4.3.2) and GraphPad Prism (version 8.0.2). Data analysis was performed using R studio (version 4.3.3), GraphPad Prism (version 8.0.2), and EXCEL software. P-values were calculated, and a P-value < 0.05 was considered statistically significant.

## 3 Results

### 3.1 Annotation, characterization, and comparative evolutionary analysis of bat IG loci

#### 3.1.1 Structure and nomenclature of bat IG H/K/L loci

The *Antrozous pallidus* IGH locus is located on chromosome 8 (CM050515.1), and the IGL locus on chromosome 21 (CM050528.1). No complete IGK locus was identified in *Antrozous pallidus.* In *Rhinolophus ferrumequinum*, the IGL locus is located on chromosome 25 (CM014250.1), the IGK locus on chromosome 13 (CM014238.1), and the IGH locus on chromosome 6 (CM014231.1) (27). The lengths of the *Antrozous pallidus* IGH and IGL loci are approximately 130 kb and 22 kb, respectively. The *Rhinolophus ferrumequinum* IGL and IGK loci are approximately 102 kb and 26 kb in length, respectively, and the IGH locus is 350 kb (27). Marked differences in both locus length and gene number were observed between the IGL loci of *Antrozous pallidus* and *Rhinolophus ferrumequinum.*

**Figure 1:**
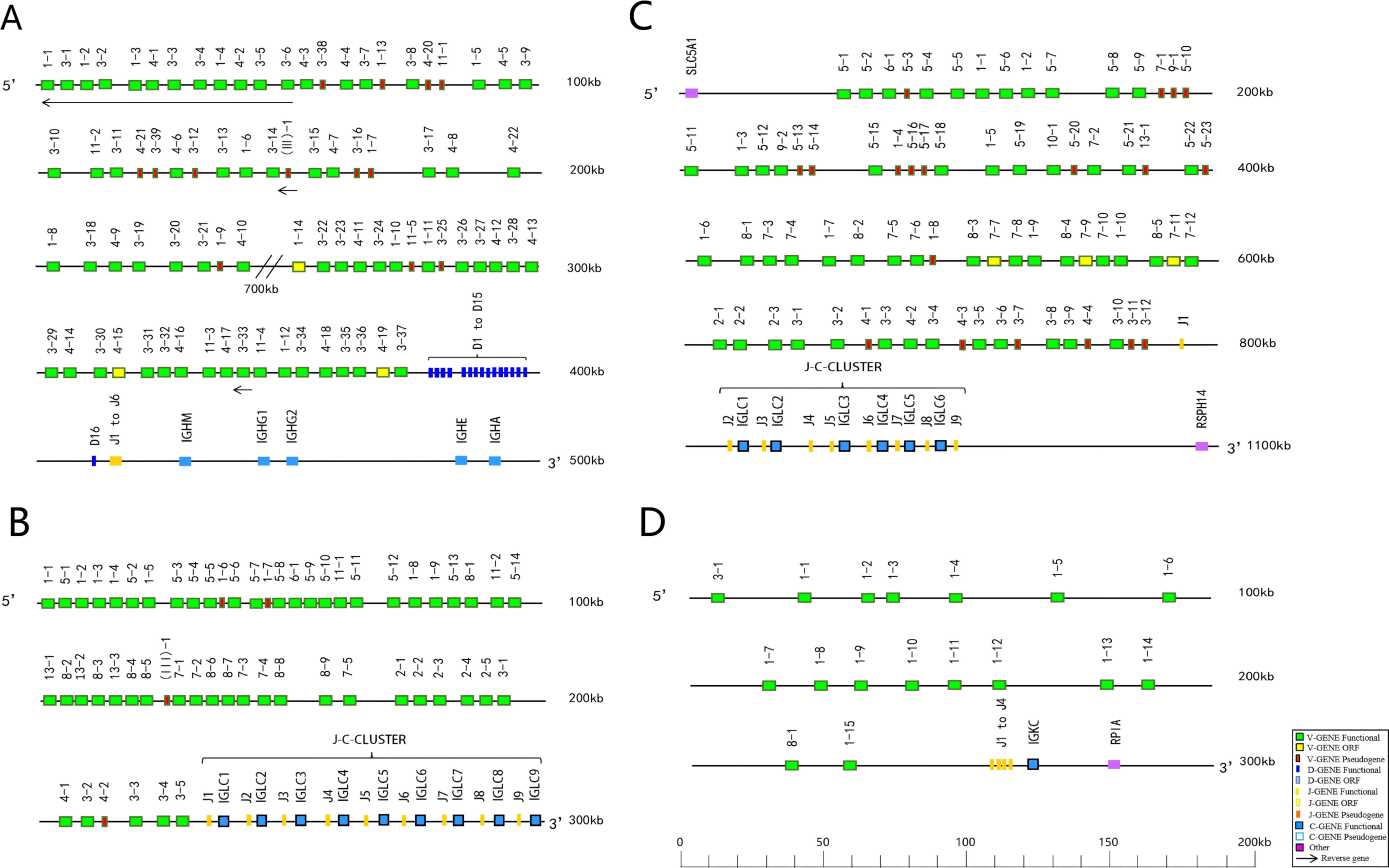
Schematic annotation of *Antrozous pallidus* IGH/IGL and *Rhinolophus ferrumequinum* IGL/IGL locus. **(A)** Schematic annotation of IGH locus in *Antrozous pallidus*. **(B)** Schematic annotation of IGL locus in *Antrozous pallidus*. **(C)** Schematic annotation of IGL locus in *Rhinolophus ferrumequinum*. **(D)** Schematic annotation of IGK locus in *Rhinolophus ferrumequinum*.

In the *Antrozous pallidus* IGH locus, 81 IGHV genes (including 14 inverted IGHV genes), 16 IGHD genes, 6 IGHJ genes, and IGHC region genes encoding IgM, IgG, IgE, and IgA were identified. However, no IgD gene was found. In the IGL locus, 56 IGLV genes and 9 J-C gene clusters were identified. In the *Rhinolophus ferrumequinum* IGL locus, 74 IGLV genes and 6 J-C gene clusters were identified. In the IGK locus, 17 IGKV genes, 4 IGKJ genes, and 1 IGKC gene were identified (**Figures 1A–D**).

According to IMGT nomenclature rules, the *Antrozous pallidus* IGHV genes are divided into 5 gene families, including 13 pseudogenes and 3 open reading frames (ORFs). *Antrozous pallidus* IGLV genes are divided into 11 gene families, including 4 pseudogenes. *Rhinolophus ferrumequinum* IGLV genes are divided into 11 gene families, including 19 pseudogenes and 3 ORFs. *Rhinolophus ferrumequinum* IGKV genes are divided into 3 families, all of which are functional genes (**Supplementary Tables 4**–**6**). The *Rhinolophus ferrumequinum* IGKV genes are predominantly clustered in the IGKV1 family. The number of IGHV pseudogenes in *Antrozous pallidus* is higher than that in *Rhinolophus ferrumequinum* (5 pseudogenes). In contrast, the number of IGLV pseudogenes in *Antrozous pallidus* is lower than that in *Rhinolophus ferrumequinum*. No pseudogenes were identified in the *Rhinolophus ferrumequinum* IGK locus (**Supplementary Tables 7**, **8**). For IGHJ, 6 gene families were named in *Antrozous pallidus.* For IGLJ, 9 and 6 gene families were named in *Antrozous pallidus* and *Rhinolophus ferrumequinum*, respectively. For IGKJ, 4 gene families were named in *Rhinolophus ferrumequinum*, all of which are functional genes (**Supplementary Figure 2**). The number of annotated heavy and light chain V(D)J genes in bats differs markedly from that in other animals; notably, the number of IGKV genes in *Rhinolophus ferrumequinum* is significantly lower than that in other mammals (**Supplementary Tables 9**–**11**).

According to the IMGT rules for mammalian IG gene organization, the amino acid structures of IGHV/IGLV/IGKV genes in *Antrozous pallidus* and *Rhinolophus ferrumequinum* all contain the same canonical conserved residues in the three framework regions: Cys (C) at position 23, Trp (W) at position 41, and Cys (C) at position 104 (**Supplementary Figures 3A–D**). The amino acid sequences of *Antrozous pallidus* IGHJ genes show a high degree of consistency with human IGHJ genes in both number and characteristics, containing the conserved WGQG motif. Most IGLJ genes in both *Antrozous pallidu*s and *Rhinolophus ferrumequinum* possess the conserved FGGG motif, with occasional mutations at individual positions. The four IGKJ gene sequences in *Rhinolophus ferrumequinum* all contain the conserved FGQG motif (**Supplementary Figure 2**).

#### 3.1.2 Evolutionary characterization of bat IG H/K/L genes and RSS sequences

**Figure 2:**
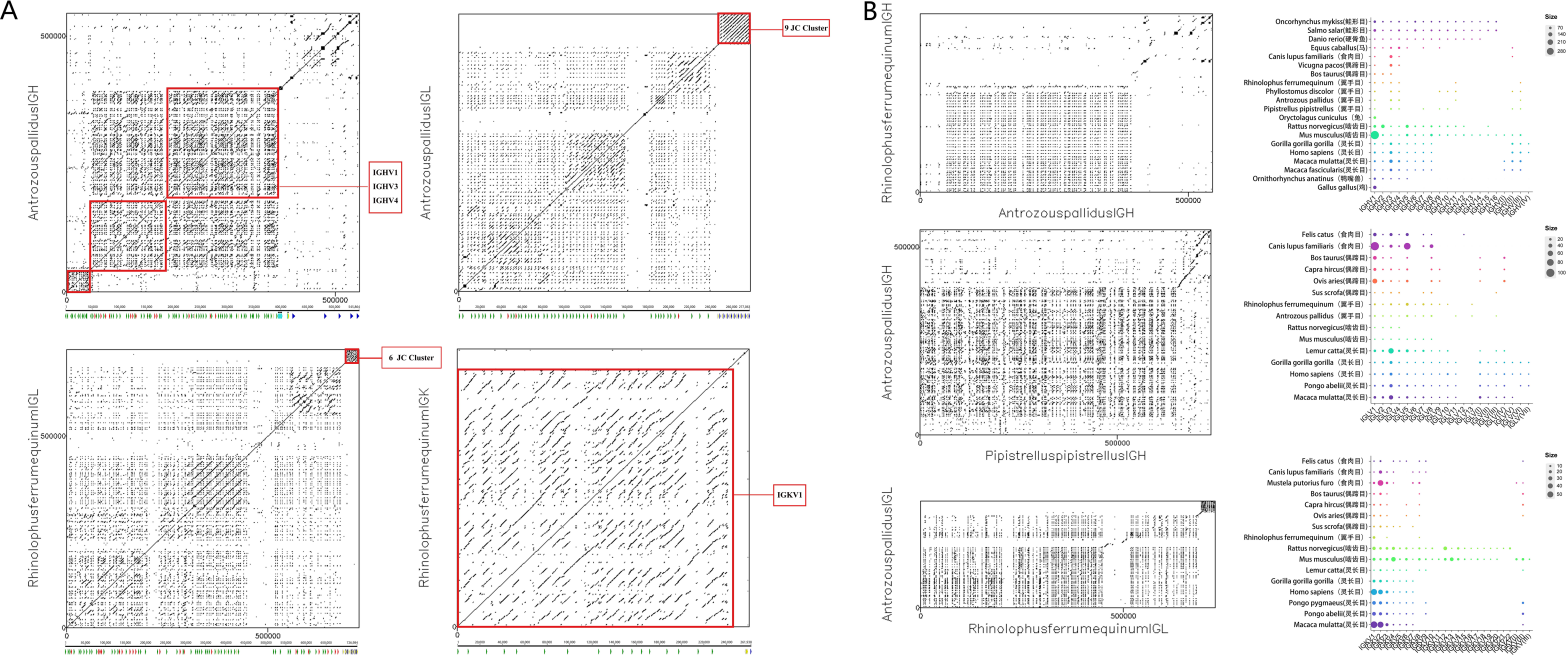
Comparison of linear analysis of IGH,IGL and IGK gene duplication in bats and the number of gene families in other species. **(A)** Linear analysis of IGH and IGL gene duplication in *Antrozous pallidus* and IGL/IGK gene duplications in *Rhinolophus ferrumequinum*. **(B)** Comparison of gene duplications in *Antrozous pallidus*, *Rhinolophus ferrumequinum* and *Pipistrellus pipistrellus*, as well as the number of gene families of the IGH, IGL, and IGK gene families in other species.

**Figure 3:**
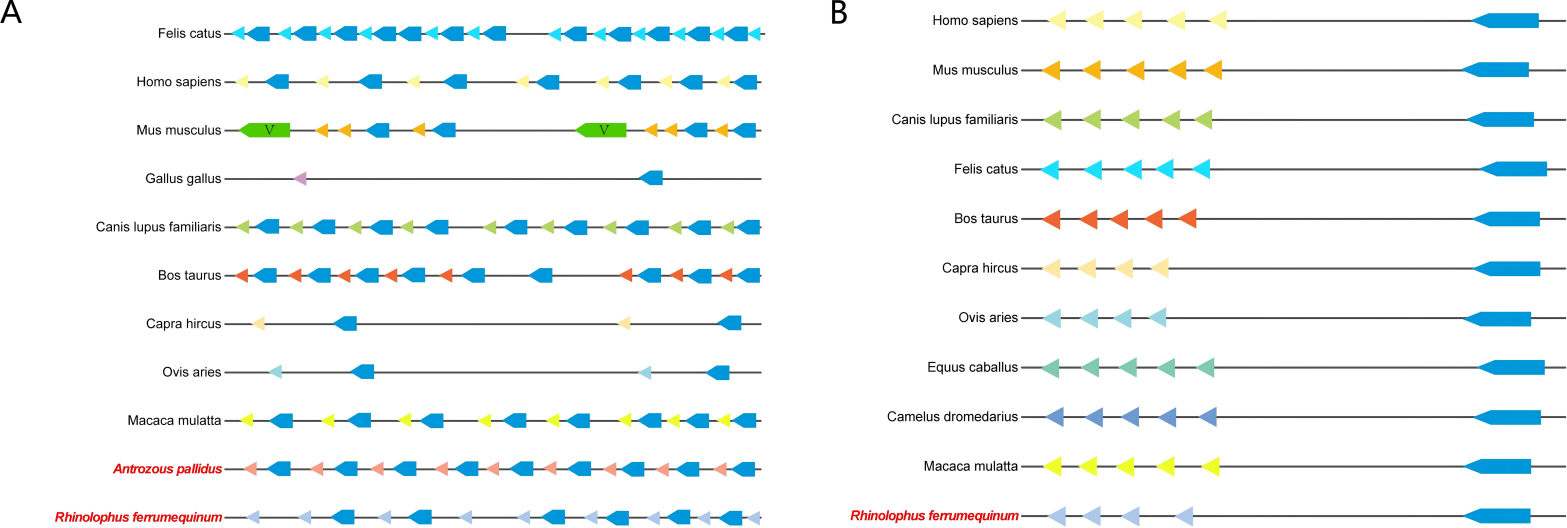
Statistical comparison of the number of J and C gene clusters in the IGL and IGK loci of bats as well as other species. **(A)** Bats and other species IGL J and C gene clusters. **(B)** Bats and other species IGK J and C gene clusters (small triangles of different colors in the figure represent J genes, blue polygons represent C genes, different colors represent different species).

In Antrozous pallidus, IGHV exhibits regional family duplication across IGHV1/3/4. The IGL gene duplication patterns are essentially identical between *Antrozous pallidu*s and *Rhinolophus ferrumequinum* (**Figure 2A**). Overall, in both *Antrozous pallidus* and *Rhinolophus ferrumequinum*, the multiple J-C gene clusters in the IGL locus are the result of gene duplication, with nine clusters in each species. The number of IGLC gene duplications is higher in *Antrozous pallidus* than in *Rhinolophus ferrumequinum*. The number of J-C gene clusters in the IGL locus varies between these two bat species and other mammals. Rodents possess two J-C clusters. Among artiodactyls, sheep and goats have two J-C clusters, while cattle have nine. Among carnivores, cats have twelve J-C clusters and dogs have nine. Among primates, humans have seven J-C clusters and rhesus macaques have eight. Chickens, as non-mammals, differ markedly from mammals, possessing only one J-C cluster (**Figure 3A**). The number of J-C clusters in the IGK locus is the same (one) between *Rhinolophus ferrumequinum* and other species, and the number of IGKJ genes is broadly similar (4–5) (**Figure 3B**). The IGH locus is shorter in *Rhinolophus ferrumequinum* than in *Antrozous pallidus*, whereas the IGH locus of *Pipistrellus kuhlii* is longer than that of *Antrozous pallidus.* The IGL locus is longer in *Rhinolophus ferrumequinum* than in *Antrozous pallidus*, and both exhibit similar patterns of gene family duplication (**Figure 2B**). Among IMGT-annotated mammals, rodents have a significantly higher number of IGHV1 genes than other mammals; carnivores and artiodactyls have more IGLV1 family genes than chiropterans and rodents; primates have more IGLV3 family genes, and the numbers are comparable among primates; primates also have higher numbers of IGKV1/IGKV2 genes than artiodactyls and chiropterans (**Figure 2B**).

**Figure 4:**
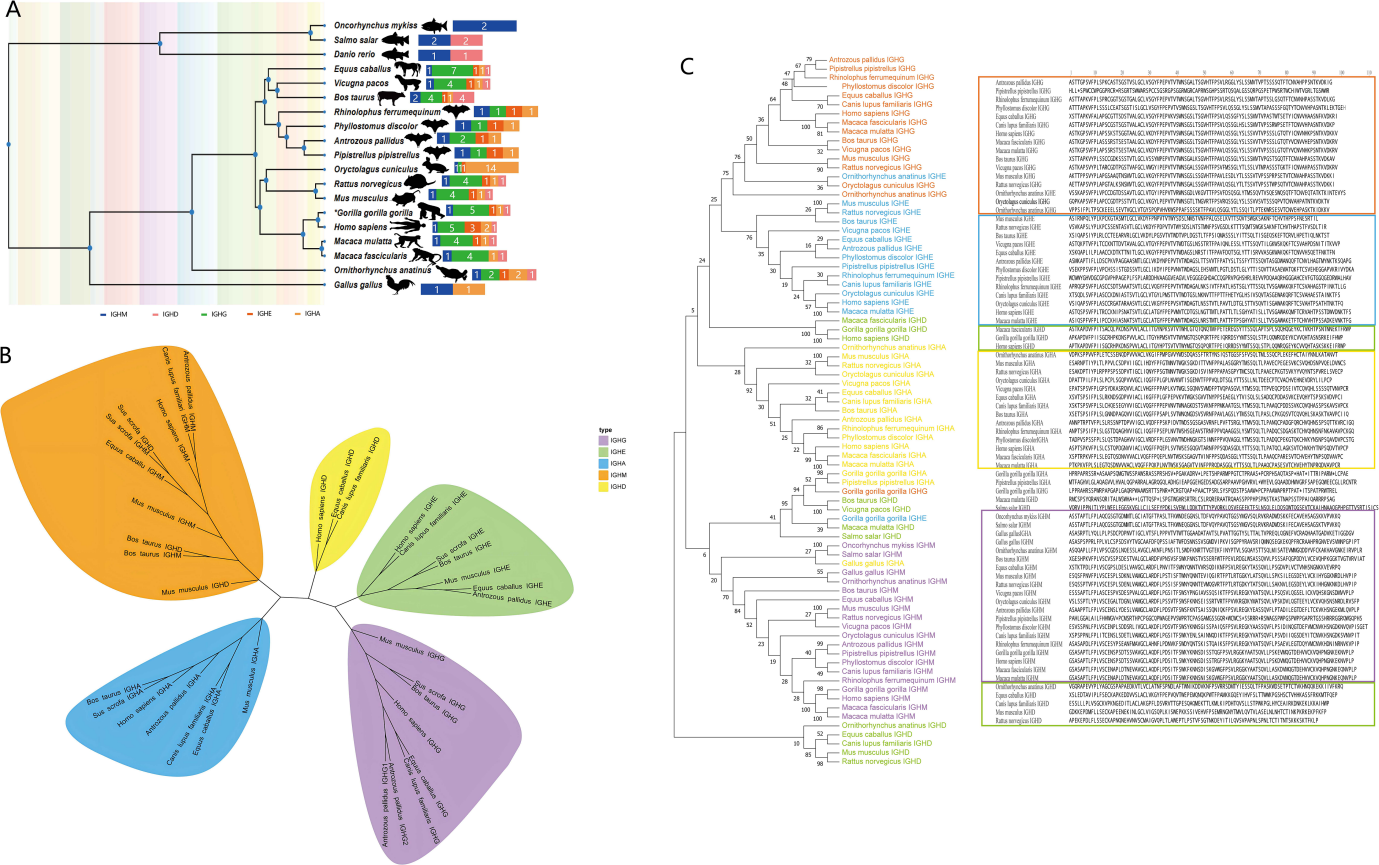
Comparative analysis of evolutionary development of IGHC genes in bats as well as other species. **(A)** Evolutionary relationship tree between bats and other species and the number of IGHC subclasses. **(B)** Evolutionary tree of IGHC subclasses between pallid cave bats and humans, dogs, cows, pigs, horses, mice. **(C)** Evolutionary tree of IGHC subclasses between pallid cave bats, martyred chrysomelid bats, pallid spear-nosed bats, and Kuhn’s bat, and other animals, and the amino acid comparisons.

*Rhinolophus ferrumequinum*, *Phyllostomus discolor*, *Pipistrellus kuhlii*, and *Antrozous pallidus* occupy an intermediate position in the species phylogenetic tree. The types and numbers of IGHC subclasses are largely similar across bats: one IgM, one IgG, one IgE, and one IgA. All lack IgD, although *Antrozous pallidus* possesses two IgG genes. Salmoniformes and teleost fish have 1 or 2 IgM and 1 or 2 IgD. Chickens have one IgM and one IgD. Rabbits differ markedly from other species, possessing 14 IgA genes. Among artiodactyls, cattle have four IgD (more than alpacas and other animals) and two IgM. Other mammals have only one IgM. Primates, rodents, artiodactyls, and horses have higher numbers of IgG genes than other IGHC subclasses, with horses having seven IgG genes in particular (**Figure 4A**).

*Antrozous pallidus* clusters with human, mouse, dog, cattle, and pig into five IGHC subclasses (**Figure 4B**). IGHC subclasses from other species also largely cluster with bat IGHC subclasses, and the amino acid sequences of the same IGHC subclass are highly similar across species (**Figure 4C**). The homology of bat heavy and light chain V genes with those of human, mouse, dog, and cattle varies. The number of IGKV gene family subclasses and genes in *Rhinolophus ferrumequinum* is far lower than that in humans and mice (**Supplementary Figures 4A, B**).

The heptamer and nonamer sequences within the IGHV 23RSS of Antrozous pallidus are relatively conserved (**Supplementary Figure 5A**). In the 23RSS upstream of IGLV genes in both Antrozous pallidus and Rhinolophus ferrumequinum, the heptamer nucleotides CAC and GTG are relatively conserved, and the nonamer nucleotides AA and C are relatively conserved, although nucleotide position 4 shows weaker conservation (**Supplementary Figure 5B**). The heptamer of the IGLJ 12RSS in *Antrozous pallidus* and *Rhinolophus ferrumequinum* shares the same conserved sites as the IGLV heptamer, and the nonamer is also relatively conserved. The GTG conservation at positions 1, 2, and 3 of the IGLJ heptamer is lower in humans and mice than in the two bat species. The human IGLJ nonamer shows similar conservation to that of the two bats, whereas the mouse IGLJ nonamer exhibits low conservation. In *Rhinolophus ferrumequinum*, both the heptamer and nonamer of the IGKV 12RSS are relatively conserved; in the IGKJ 23RSS, the heptamer nucleotides GTGTC are relatively conserved, but the nonamer shows low conservation at position 9 (**Supplementary Figure 5C**).

### 3.2 Characteristics of the bat BCR CDR3 repertoire and comparative analysis with humans and mice

#### 3.2.1 General characteristics of the bat BCR CDR3 repertoire

**Figure 5:**
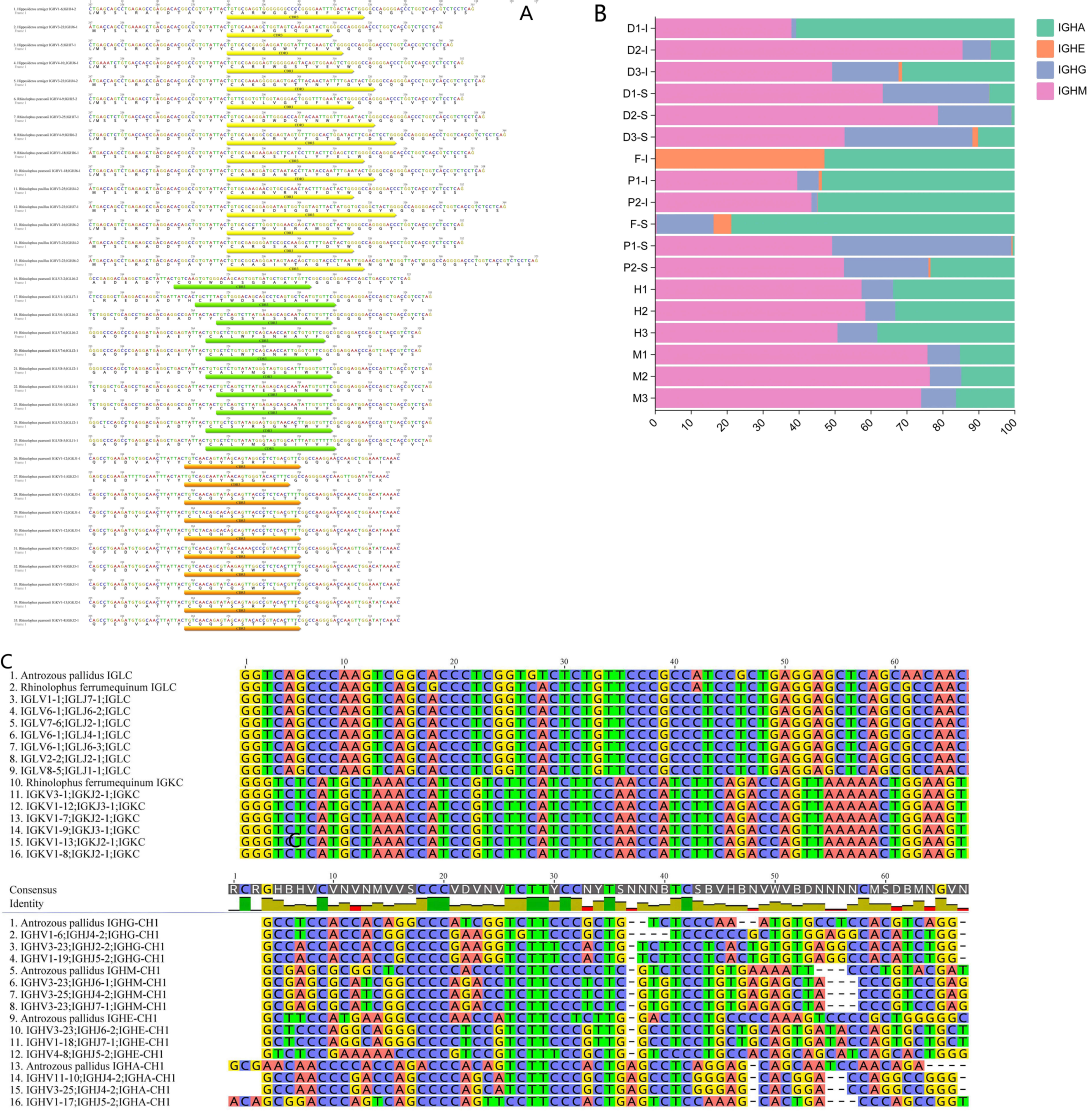
Examples of bat IGH/IGL/IGK V-J-C gene accessions and the percentage of IGHC subclasses in bat, human and mouse samples. **(A)** Example of IGH/IGL/IGK V-J gene usage in bats. **(B)** Percentage of IGHC subclasses in bat, human and mouse samples; **(C)** Example of IGH/IGL/IGK C gene usage in bats.

Quality control of BCR CDR3 library construction from bat spleen and intestinal samples showed a prominent peak at the 1500 bp target band, indicating successful library construction. The total number of functional BCR CDR3 sequences and unique sequences varied considerably among the 10 bats (**Table 1**), but all met the basic requirements for HTS analysis of the CDR3 repertoire. Human and mouse BCR CDR3 sequences (**Supplementary Tables 2**, **3**) were included for comparative analysis. It was found that the IGH CDR3 repertoire in both spleen and intestine of *Hipposideros armiger* was dominated by IgM, whereas in *Rhinolophus pearsonii*, the intestinal IGH CDR3 was dominated by IgG and the splenic IGH CDR3 by IgM. In sample F-I, IgE accounted for a relatively high proportion, while in sample F-S, IgG accounted for a high proportion. In contrast, the IGH CDR3 repertoire in both humans and mice was dominated by IgM (**Figure 5B**). The numbers of IGL/IGK CDR3 sequences in bats were relatively low, showing high consistency with humans and mice. The IGH CDR3 region sequence composition of *Hipposideros armiger, Rhinolophus pearsonii*, and *Rhinolophus pusillus* indicated high homology between the families *Rhinolophidae* and Hipposideridae (**Figure 5A**). The annotated C regions of *Antrozous pallidus and Rhinolophus ferrumequinum* showed high homology with those of *Hipposideros armiger* and *Rhinolophus pearsonii* (**Figure 5C**).

**Table 1:** Bat spleen and gut IGH/K/L CDR3 HTS data-sheet.

Bat	Name	Type	Sample	Productive	Clonotype
*Hipposideros armiger*	IGH	Spleen	D1-S	3033398	24198
		Spleen	D2-S	2232406	39347
		Spleen	D3-S	2529443	20739
*Hipposideros armiger*	IGH	Intestine	D1-I	2545350	8842
		Intestine	D2-I	3194348	14754
		Intestine	D3-I	201539	1136
*Rhinolophus*	IGH	Spleen	F-S(*pusillus)*	24649	591
		Spleen	P1-S*(pearsonii)*	673914	25817
		Spleen	P2-S*(pearsonii)*	1788	700
*Rhinolophus*	IGH	Intestine	F-I(*pusillus)*	130615	318
		Intestine	P1-I*(pearsonii)*	8644	1271
		Intestine	P2-I*(pearsonii)*	70242	4098
*Rhinolophus pearsonii*	IGH	Spleen	P1-IGH	206024	9526
			P2-IGH	261244	8500
			P3-IGH	741592	27783
			P4-IGH	1050313	45558
*Rhinolophus pearsonii*	IGL	Spleen	P1-IGL	10384591	18553
			P2-IGL	16157961	26373
			P3-IGL	11479657	72629
			P4-IGL	13177606	58719
*Rhinolophus pearsonii*	IGK	Spleen	P1-IGK	4800633	2685
			P2-IGK	7194742	3847
			P3-IGK	4123972	6150
			P4-IGK	5426366	7141

#### 3.2.2 Characteristics of the bat IGH CDR3 repertoire and comparative analysis

**Figure 6:**
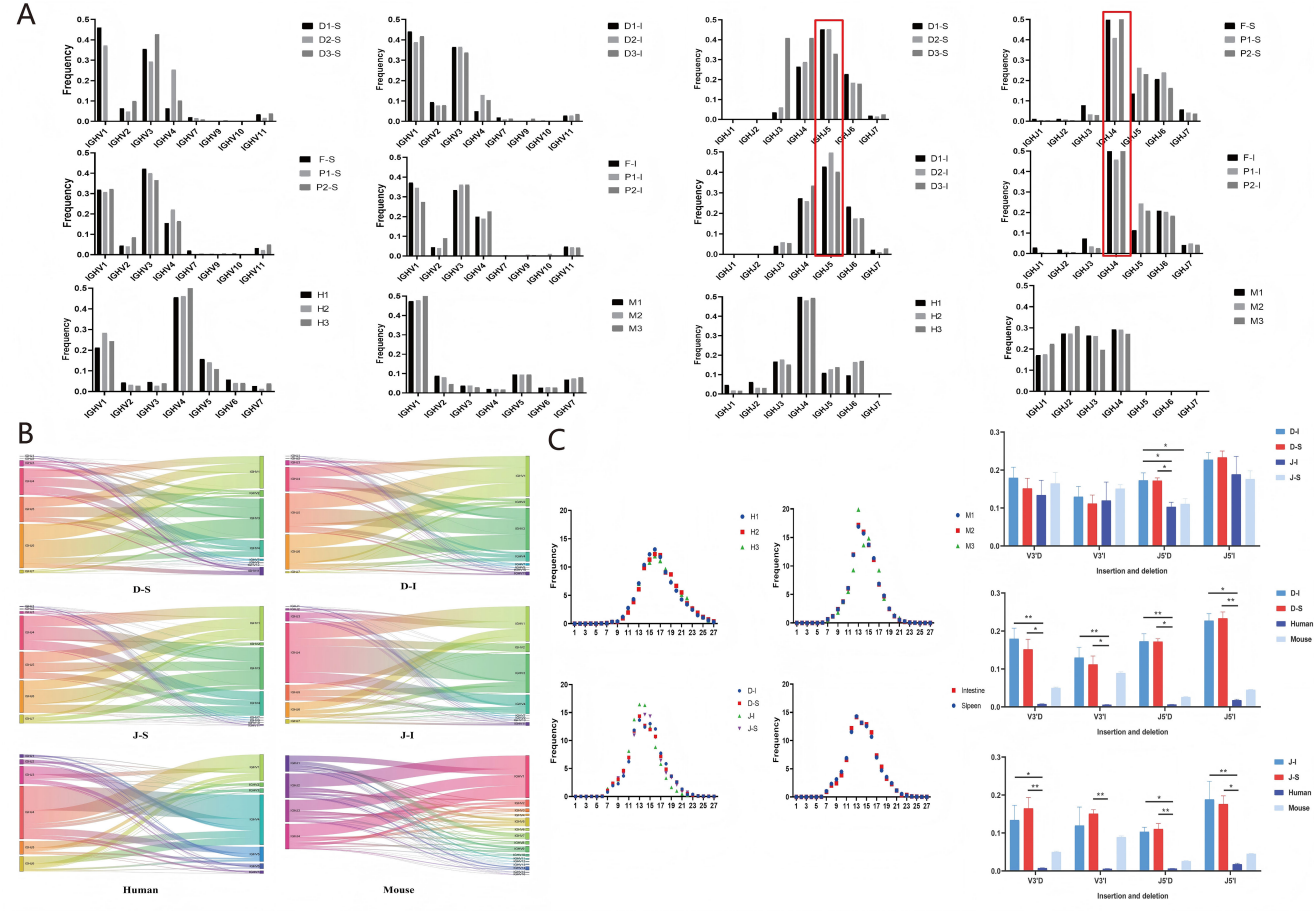
Analysis of bat spleen and intestine, human peripheral blood, and mouse bone marrow IGHV-IGHJ gene fetch and pairing, and IGH CDR3 length and shear insertion analysis (note: The IGH CDR3 repertoires data from normal mice and healthy volunteers used in this study for comparative analysis were obtained from the same sources as those used in our previously published article (Liu Ma L et al., Reference 27).) **(A)** Bat, human, and mouse IGHV/IGHJ gene fetch. **(B)** Bat, human, and mouse IGHV-IGHJ gene pairing; **(C)** Bat, human, and mouse IGH CDR3 length distribution as well as shear and insertion analyses (Kruskal-Wallis H Test, P < 0.05, *:indicates P < 0 .05, **:indicates P < 0 .01).

IGH CDR3 V and J usage and V-J pairing: *Hipposideros armiger, Rhinolophus pearsonii*, humans, and mice all showed high-frequency usage of IGHV1, while the two bat species uniquely showed high-frequency usage of IGHV3. In *Hipposideros armiger*, IGHJ5 was the most frequently used in both spleen and intestine, whereas *Rhinolophus pearsonii* showed high-frequency usage of IGHJ4. IGHJ gene usage in *Rhinolophus pearsonii* was closer to that in humans. IGHV and IGHJ usage in humans and mice were consistent with previous studies (**Figure 6A**). In *Hipposideros armiger*, the predominant high-frequency V-J pairing was IGHV1-IGHJ5 in both spleen and intestine, while in *Rhinolophus pearsoni*i, the predominant high-frequency pairing was IGHV1-IGHJ4. *Rhinolophus pearsonii* shared the same high-frequency pairing as humans, whereas mice predominantly showed high-frequency pairings of IGHV1 with four IGHJ families (**Figure 6B**). The trend of V-J pairing in individual samples from spleen and intestine was consistent between *Hipposideros armiger* and *Rhinolophus pearsonii* (**Figure 6B**).

IGH CDR3 length distribution, N/P nucleotide Additions/Deletions: In both *Hipposideros armiger* and *Rhinolophus pearsonii*, the CDR3 length distribution in spleen and intestine peaked at 13 amino acids (AA). Mice also showed a peak at 13 AA, whereas humans showed a peak at 16 AA. The nucleotide deletion at the J 5′ end was higher in *Hipposideros armige*r than in *Rhinolophus pearsonii* in both spleen and intestine, but no significant differences in nucleotide insertion and deletion were observed between spleen and intestine within either bat species. Nucleotide deletion and insertion at the V 3′ end and J 5′ end were higher in both bat species than in humans and mice (**Figure 6C**). The CDR3 length distribution in individual samples from spleen and intestine showed the same trend between *Hipposideros armiger* and *Rhinolophus pearsonii* (**Supplementary Figure 7**).

**Figure 7:**
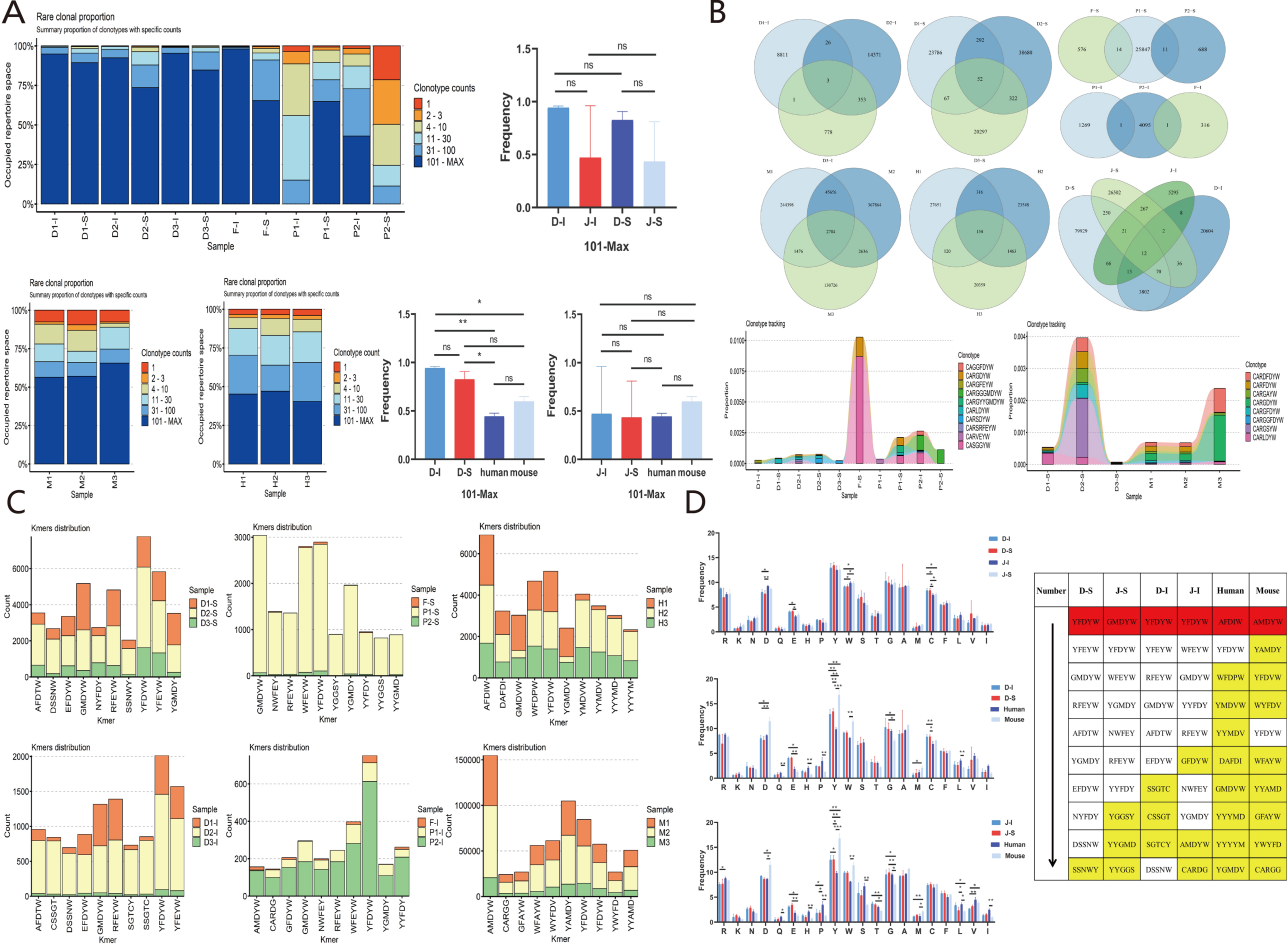
Bat spleen and intestine, human peripheral blood, mouse bone marrow IGH CDR3 rare clone frequency distribution, overlap and clone frequency tracking analysis, top 10 HF Motif and AA fetch analysis. **(A)** Bat, human, and mouse IGH CDR3 rare clone frequency distribution. **(B)** Bat, human, and mouse IGH CDR3 overlap analysis and clone frequency tracking. **(C)** Bat, human, and mouse IGH CDR3 top 10 high-frequency motif analysis. **(D)** Bat, human, and mouse IGH CDR3 AA access analysis (Kruskal-Wallis H Test; one- way analysis of variance, P < 0.05, *:indicates P < 0 .05, **:indicates P < 0 .01, ***:indicates P < 0 .001).

IGH CDR3 diversity and clonality: The diversity in spleen and intestine was higher in *Hipposideros armiger* than in *Rhinolophus pearsonii*, and both bat species showed higher diversity than humans and mice, with a marked difference from mice (**Supplementary Figure 8**). Defining rare clones as those with fewer than 100 CDR3 sequences, *Hipposideros armiger* showed a relatively low proportion of rare clones in spleen and intestine, whereas *Rhinolophus pearsonii* P1-I and P2-S had higher proportions of rare clones. No significant differences were observed in CDR3 clonotypes with >101 sequences between *Hipposideros armiger* and *Rhinolophus pearsonii* in spleen and intestine. The proportion of rare clonotypes was higher in both bat species than in humans and mice. The proportion of CDR3 clonotypes with >101 sequences in the intestine of *Hipposideros armiger* differed from that in humans and mice (**Figure 7A**). No significant differences were observed in high-frequency CDR3 clones between the intestine and spleen of *Hipposideros armiger* and *Rhinolophus pearsonii* (**Supplementary Figure 9**).

IGH CDR3 overlap analysis and clonal tracking: In *Hipposideros armiger*, 52 unique overlapping sequences were found in the spleen, but only 3 unique overlapping sequences in the intestine. In *Rhinolophus pearsonii*, only samples P1-S and P2-I shared unique overlapping sequences with other samples. The number of unique overlapping sequences was higher in humans and mice than in the two bat species. A total of 12 overlapping sequences were found between spleen and intestine across *Hipposideros armiger* and *Rhinolophus pearsonii;* clonal tracking of these 12 sequences revealed that 10 could be tracked across all 10 samples. Overlapping sequences were found between the spleen of *Hipposideros armiger* and mouse (**Figure 7B**). One shared overlapping sequence, CARDYYGMDYW, was found between spleen and intestine in *Hipposideros armiger*, but none in *Rhinolophus pearsonii* (**Supplementary Figure 10**). Overlap index analysis showed no difference between spleen and intestine within *Hipposideros armige*r and *Rhinolophus pearsonii*, but differences were observed between *Rhinolophus pearsonii* spleen/intestine and mouse, and between *Rhinolophus pearsoni*i intestine and human (**Supplementary Figure 11**).

IGH CDR3 motif composition and AA usage analysis: The most frequent motif in the spleen and intestine of *Hipposideros armiger* and the intestine of *Rhinolophus pearsonii* was consistently YFDYW, whereas another most frequent motif, GMDYW, appeared in the spleen of *Rhinolophus pearsonii.* The most frequent motifs in humans and mice were AFDIW and AMDYW, respectively. Both bat species, along with humans and mice, shared a high-frequency motif, YFDYW (**Figures 7C, D**). In *Hipposideros armiger* and *Rhinolophus pearsonii*, the amino acids Y, G, A, R, W, D, and C were frequently used in both spleen and intestine, with significant differences from humans and mice in the usage of Y, W, D, H, and E. Tyrosine (Y) was the dominantly used amino acid in bats, humans, and mice. No significant difference in tyrosine usage was observed between spleen and intestine within the two bat species, but significant differences were observed between bats and humans/mice (**Figure 7D**).

#### 3.2.3 Characteristics of the bat IGK/L CDR3 repertoire and comparative analysis

**Figure 8:**
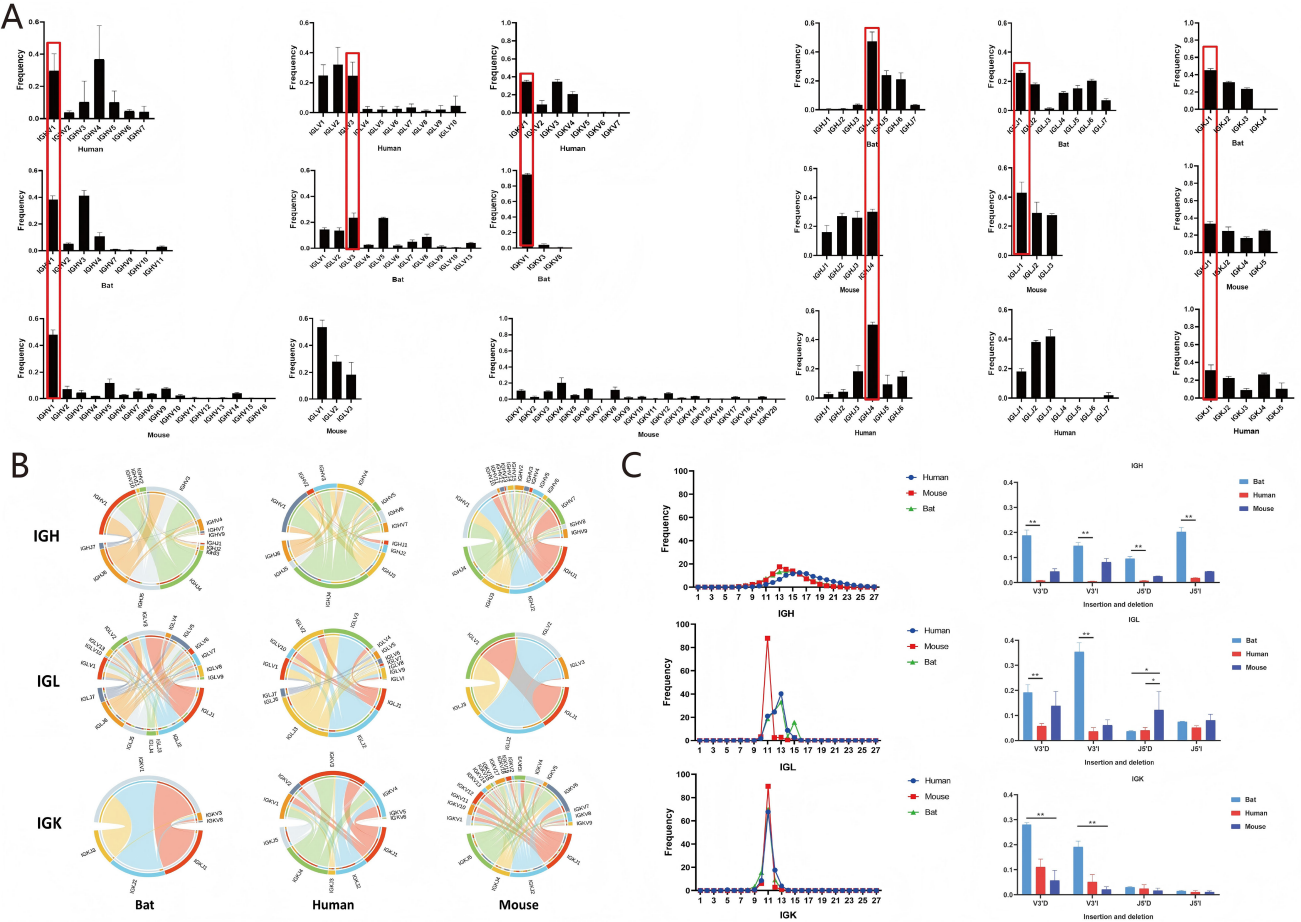
Bat, human, and mouse IGH/IGL/IGK V-J gene fetch and pairwise analysis and CDR3 length distribution and shear insertion analysis. **(A)** Bat, human, and mouse IGH/IGL/IGK V-J gene fetch. **(B)** Bat, human, and mouse IGH/IGL/IGK V-J gene pairing. **(C)** Bat, human, and mouse IGH/IGL/IGK CDR3 length distribution and shear insertion analyses (Kruskal-Wallis H Test, P < 0.05, *:indicates P < 0.05, **:indicates P < 0.01).

IGK/L CDR3 V and J usage and V-J pairing: For IGL, bats and humans showed high frequency usage of IGLV3, while mice showed high-frequency usage of IGLV1, bats uniquely showed high-frequency usage of IGLV5. For IGK, both bats and humans showed high-frequency usage of IGKV1, whereas mice showed high-frequency usage of IGKV4 and IGKV6. Bats and mice showed high-frequency usage of IGLJ1, while humans showed high-frequency usage of IGLJ3. For IGK, bats, humans, and mice all showed high-frequency usage of IGKJ1 (**Figure 8A**). For IGL, the predominant high-frequency V-J pairing was IGLV3-IGLJ1 in bats, IGLV3-IGLJ3 in humans, and IGLV1-IGLJ1 in mice. For IGK, the predominant high-frequency pairing was IGKV1-IGKJ1 in bats; humans showed high-frequency pairings of IGKV1 and IGKV3 with IGKJ1 and IGKJ4; mice showed high-frequency pairings of IGKV4/IGKV6 with IGKJ1/J2/J4/J5 (**Figure 8B**). The trend of V-J pairing in individual bat samples was consistent (**Supplementary Figure 12**).

IG L/K CDR3 length distribution, N/P nucleotide Additions/Deletions: For IGL, the CDR3 length distribution peaked at 13 AA in both bats and humans, with bats additionally showing a second peak at 15 AA; mice peaked at 11 AA. For IGK, bats, humans, and mice all showed a peak at 11 AA (**Figure 8C**). Nucleotide insertion and deletion at the V 3′ end of IGL were higher in bats than in humans and mice, with a significant difference from humans. Nucleotide insertion and deletion at the J 5′ end were lower in bats and humans than in mice, with significant differences. For the IGK chain, nucleotide insertion and deletion at both the V 3′ and J 5′ ends were higher in bats than in mice and humans (**Figure 8C**). The trend of CDR3 length distribution in individual samples was consistent across bats, humans, and mice (**Supplementary Figure 13**).

**Figure 9:**
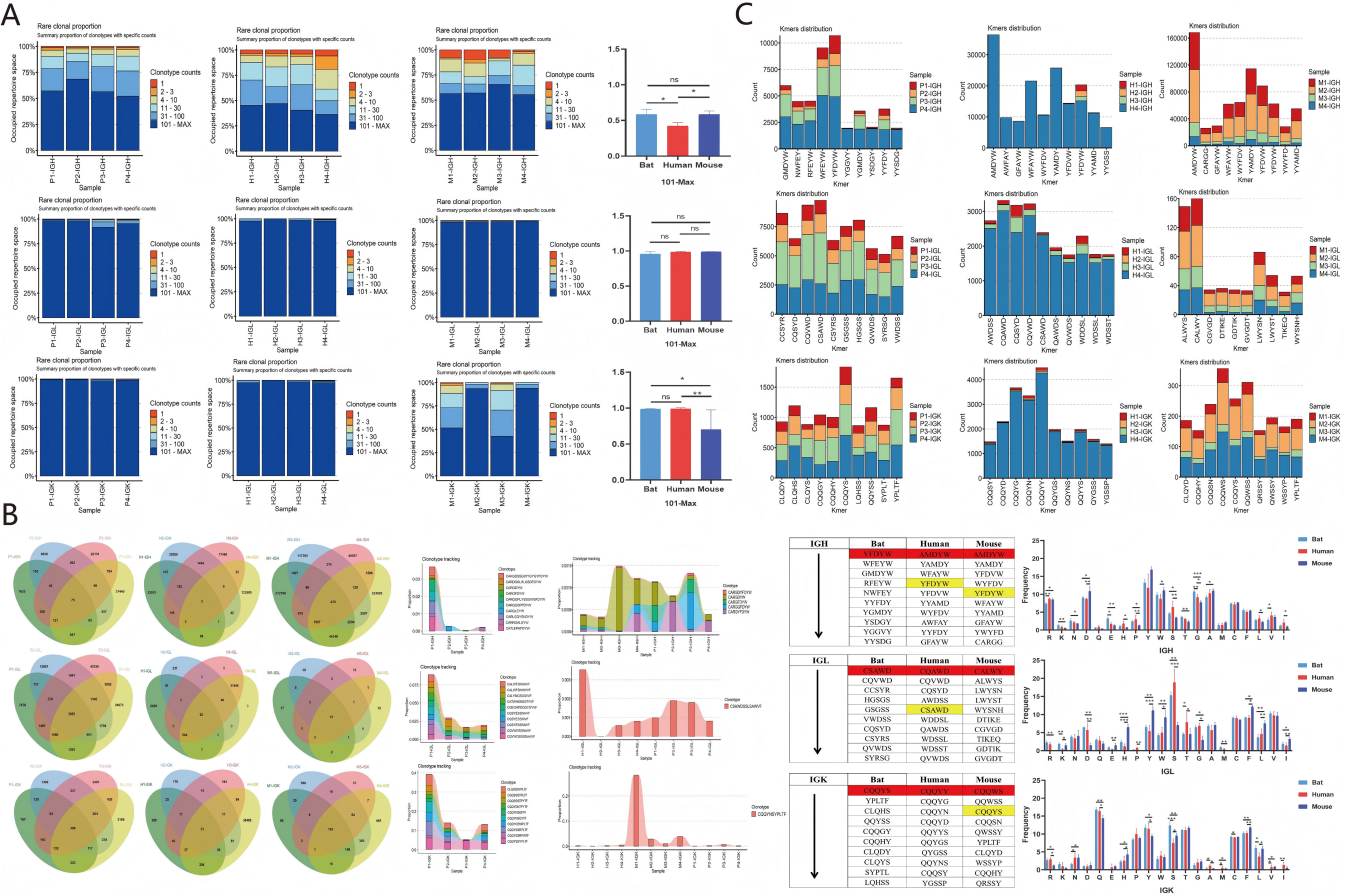
Bat, human, and mouse IGH/IGL/IGK CDR3 rare clone frequency distribution, overlap and clone frequency tracking analysis, top 10 high frequency Motif and AA fetch analysis. **(A)** Frequency distribution of rare clones in bat, human, and mouse IGH/IGL/IGK CDR3. **(B)** Overlap analysis and clone frequency tracking in bat, human, and mouse IGH/IGL/IGK CDR3. **(C)** Analysis of the top 10 high-frequency Motif and AA fetching in bat, human, and mouse IGH/IGL/IGK CDR3 (Kruskal-Wallis H Test, one-way analysis of variance, P < 0.05, *:indicates P < 0.05, **:indicates P < 0.01, ***:indicates P < 0.001).

IG L/K CDR3 diversity and clonality: IGL CDR3 diversity was higher in bats than in humans and mice, whereas IGK CDR3 diversity was lower in bats than in humans and mice (**Supplementary Figure 14**). The proportion of rare IGL CDR3 clones was very low in bats, humans, and mice. For IGK CDR3, the proportion of clonotypes with >101 sequences was very high in bats and humans (**Figure 9A**). Marked differences in IGL CDR3 clonality were observed among bats, humans, and mice (**Supplementary Figure 15**).

IG L/K CDR3 overlap analysis and clonal tracking: The number of unique overlapping CDR3 sequences for both IGL and IGK was higher in bats than in humans and mice. The top 10 shared high-frequency CDR3 clones could be tracked across all samples, with the highest proportion in sample P1 and the lowest in sample P3. Bats, humans, and mice shared one identical IGK CDR3 clone, CQQYNSYPLTF (**Figure 9B**). Significant differences in the overlap index for IGL and IGK CDR3 were observed among mice, bats, and humans (**Supplementary Figure 16**).

IG L/K motif composition and AA usage comparison: Humans shared a high-frequency IGL motif (CSAWD) with bats. Mice and bats shared the same high-frequency IGK motif (CQQYS) (**Figure 9C**). For IGL, bats, humans, and mice all showed high-frequency usage of the amino acids S, W, Y, C, F, and V. Among these, mice showed the highest frequency of Y, whereas bats and humans showed the highest frequency of S; the amino acid usage in bats was closer to that in humans than to that in mice. For IGK, bats, humans, and mice all showed high-frequency usage of the amino acids Q, P, Y, S, T, C, and F. The overall trend of IGK amino acid usage was similar among bats, humans, and mice (**Figure 9C**).

## 4 Discussion

The mechanisms by which bat innate immunity tolerates viruses have been extensively reported (18, 30), however, the composition of the bat adaptive immune response system and its role in viral tolerance remain poorly understood (17, 19, 31, 32). Early studies found that many bats develop neutralizing antibody responses following viral infection (33–35), suggesting that B-cell adaptive immunity may play an important role in the antiviral immune response of bats. To date, studies characterizing immunoglobulin genes in bats have focused on the families *Pteropodidae* and *Rhinolophidae* (16, 26, 27). The adaptive immune response of most bat species remains unexplored. Building on our previous successful annotation of the bat IGH locus, we screened chromosome-level assemblies of *Antrozous pallidus* and *Rhinolophus ferrumequinum*, completed the first annotation of the IG light chain loci, and performed the first HTS and comparative characterization of the BCR CDR3 repertoire in *Hipposideros armiger* and *Rhinolophus pearsonii.*

### 4.1 Annotation results and evolutionary analysis of the IGH/IGL/IGK loci in *Antrozous pallidus* and *Rhinolophus ferrumequinum*

The overall structures of the IGH/IGL/IGK loci in *Antrozous pallidus* and *Rhinolophus ferrumequinum* are conserved. The IGH locus of A*ntrozous pallidus* is similar in structure to those we previously annotated in *Rhinolophus ferrumequinu*m*, Phyllostomus discolor*, and *Pipistrellus kuhlii* (27), and also resembles the annotated IGH locus of the Egyptian fruit bat (36). However, marked differences were observed in the number of IGHV genes and the length of the IGH locus among different bat species. Similarly, the length and gene number of the IGL locus differed significantly between *Antrozous pallidus* and *Rhinolophus ferrumequinum*. Notably, a gap of over 700 kb was identified in the IGH germline V gene region of *Antrozous pallidus*, which increases the distance between some V genes and the downstream D-J region. Such gaps have also been observed in annotations of other species and may be related to genome assembly and gene distribution.

The *Antrozous pallidus* IGH locus contains 81 IGHV genes, including 14 inverted genes, suggesting that inverted genes in bats represent an important direction for functional studies. Among the fully annotated bat IGH loci to date, all lack the C region of IgD, although transcripts of IgD have been detected in insectivorous bats (16, 37). The absence of IgD may be a consequence of evolutionary selection in bats. Since no complete bat IGK and IGL loci had been annotated previously, we report here for the first time that the *Rhinolophus ferrumequinum* IGL locus contains 74 IGLV genes, including 19 pseudogenes; the *Antrozous pallidus* IGL locus contains 56 IGLV genes, including 4 pseudogenes, and all *Rhinolophus ferrumequinum* IGKV genes are functional. A complete IGK locus was not localized in *Antrozous pallidus*, consistent with the absence of a complete IGK locus in the big brown bat (38), suggesting that the expansion patterns of bat IG light chain loci are an important direction for understanding their function. Two functional IGH loci have been annotated in the big brown bat (38), but we did not observe this in *Antrozous pallidus* or in our previously annotated *Rhinolophus ferrumequinum, Phyllostomus discolor*, and *Pipistrellus kuhlii* (27). Duplicated IGH loci and loss of the IGK locus, as seen in bats, have not been observed in any of the multiple mammalian species fully annotated in IMGT, suggesting a unique evolutionary mechanism of bat IG loci that may be related to the role of bat B cells in viral tolerance. However, the absence of the IGK locus is also found in birds, which has been linked to flight-related metabolism (39–41). Although bats and birds are not the same species, they share the characteristic of flight, suggesting that the unique metabolism of flying animals may involve distinct B-cell adaptive immune evolution mechanisms.

The inconsistency in the number of IGHV gene families among different bat species (*Antrozous pallidus* = 5;*Rhinolophus ferrumequinum* = 6;Egyptian fruit bat = 4) may be due to differences in classification criteria used for annotation. However, the distribution of IGL gene families is consistent between *Antrozous pallidus* and *Rhinolophus ferrumequinum* (11 families each), which is relatively high compared with other IMGT-annotated species (human=16;mouse=8;cattle=8;dog=8;chicken=1). In contrast, the *Rhinolophus ferrumequinum* IGK gene family comprises only 3 families, fewer than in other annotated species (human=7; cattle=5; dog=9; mouse= 22). This highlights the unique evolution of bat IGL and IGK loci.

The expansion patterns of IG gene families reflect species-specific differences in immune evolution. Gene duplication generates new gene functions that enable species to better adapt to environmental changes, improving survival and reproductive fitness. Under environmental pressure, favorable gene duplications contribute to individual survival and reproduction, thereby driving species evolution and diversification and enriching population genetic diversity (42–46). We observed gene family duplication and expansion in the IGH/IGL/IGK genes of both *Antrozous pallidus* and *Rhinolophus ferrumequinum*, with *Antrozous pallidus* showing predominant expansion of the IGHV1/3/4 families and *Rhinolophus ferrumequinum* showing predominant expansion of the IGKV1 family. This suggests that the vast diversity of bat species may provide greater potential for B-cell adaptive immune responses. In our comparisons, we also found that the number of J-C clusters in artiodactyls and carnivores far exceeds that in chiropterans, indicating class-level divergence in the recombination strategy of IG light chain loci. The 12 J-C clusters in cats (Carnivora) may support their exposure to complex pathogens, whereas the simplified pattern in rodents (2 J-C clusters) may be associated with their shorter lifespan.

The constant region structures of IGHC are highly conserved across species evolution. In this study, we also found that bat IGHC subclasses cluster with those of different species, suggesting that bat antibody effector functions may rely on somatic hypermutation rather than subclass expansion. However, IGHC genes across different species have evolved more classes and more complex structures, as seen in chickens, teleost fish, and salmoniformes, reflecting the trend toward progressive refinement and complexity of the immune system during evolution.

Bat IGHV/IGLV/IGKV genes cluster with those of humans, mice, dogs, cattle, and other mammals in a family-wise manner, without showing significant homology to any single species. The V and J genes of the IG H/L loci in *Antrozous pallidus* and the IG L/K loci in *Rhinolophus ferrumequinum* are highly conserved at the amino acid level, and the lengths of IGHJ and IGLJ (approximately 38 bp) are essentially consistent with those of other mammals.

RSS sequences are key components involved in gene rearrangement and are highly conserved during evolution. We found that the RSS conservation of the IGH locus in *Antrozous pallidus* and *Rhinolophus ferrumequinum* is higher than that of the IG L/K loci, similar to the pattern in humans, whereas RSS conservation in mice shows a decreasing trend. This suggests that the participation of RSS in V(D)J recombination may differ in its effects among different mammalian species.

### 4.2 Characteristics and comparative analysis of the BCR CDR3 repertoire in *Hipposideros armiger* and *Rhinolophus pearsonii*

The V and J usage bias, diversity, clonality, overlap, and CDR3 length of the BCR CDR3 repertoire are fundamental for dissecting B-cell adaptive immune responses. However, HTS analysis of the bat BCR CDR3 repertoire remains very limited. In this study, we performed for the first time HTS and characterization of the BCR CDR3 repertoire in the spleen and intestine of *Hipposideros armiger* and *Rhinolophus pearsonii/pusillus.*

The preferential usage of V and J genes in the BCR repertoire is linked to B-cell adaptive immune responses in a species-specific manner. We previously observed differences in V and J usage among different bat species (27). In the present study, both *Hipposideros armiger* and *Rhinolophus pearsonii* showed high-frequency usage of IGHV1 and IGHV3, with the high-frequency usage of IGHV3 being markedly distinct from that in humans and mice. This suggests that the IGHV1 gene family plays a conserved core function in B-cell responses, whereas the uniquely high-frequency usage of IGHV3 in bats may represent a target of selective pressure associated with their long-term role as viral reservoirs (47). Humans predominantly use IGHJ4, whereas we found that *Hipposideros armiger* preferentially uses IGHJ5 and *Rhinolophus pearsonii* preferentially uses IGHJ4, with consistent results in both spleen and intestine. This suggests that the adaptive immune response mechanisms in bats may be more complex than in other mammals, and that preferential usage may be related to the distance between V genes (48). Similar features have been observed in other studies, such as the preferential usage of IGHJ4 in the Egyptian fruit bat (36). Such differences in usage may lead to functional divergence, for example, the high-frequency IGHV1-IGHJ5 pairing observed in *Hipposideros armiger* involves a longer IGHJ5 coding region that may increase CDR3 flexibility, potentially enabling broader antigen recognition (3). The V and J gene usage at the IGL and IGK loci annotated in the two bat species showed trends closer to those in humans and more divergent from mice, suggesting that bats and humans may have more similar B-cell response capabilities.

BCR CDR3 length is associated with B-cell response efficacy, primarily determined by nucleotide deletion and insertion during V(D)J recombination. The IGL CDR3 length distribution in bats displayed a bimodal pattern with peaks at 13 AA and 15 AA, indicating the ability to rapidly respond to a broad spectrum of antigens (shorter CDR3) as well as to target specific pathogens with high affinity (longer CDR3). Nucleotide deletion and insertion at the J 5′ end were significantly higher in *Hipposideros armiger* than in *Rhinolophus pearsonii*, potentially conferring a broader antigen response breadth in *Hipposideros armiger.* Moreover, N/P nucleotide Additions/Deletions at the IGH locus in both bat species were higher than in humans and mice, suggesting a unique advantage in bat B-cell responses.

BCR CDR3 diversity, clonality, and overlap are linked to the intensity and breadth of adaptive immune responses. The diversity of the BCR CDR3 repertoire in both the intestine and spleen of *Hipposideros armiger* was higher than that in *Rhinolophus pearsonii* and significantly higher than in humans and mice, suggesting that bat B cells may recognize a broader range of epitopes, particularly the diversity of IgA in the intestine, which is closely associated with the ability to tolerate pathogens (49). The marked differences in BCR CDR3 diversity for IGL and IGK among bats, humans, and mice represent a potential direction for further investigating species-specific B-cell response strategies (50). Additionally, we observed differences in clonal expansion frequencies in the BCR CDR3 repertoire between *Hipposideros armiger* and *Rhinolophus pearsonii*, indicating different B-cell response states (47). The identification of overlapping BCR CDR3 sequences or motifs among individuals of bats, mice, and humans provides a foundation and new insights for in-depth studies of cross-species responses to identical antigenic epitopes (51). The preliminary findings of differential amino acid usage in the BCR CDR3 region among bats, humans, and mice offer theoretical support and novel perspectives for investigating the breadth and depth of B-cell responses to antigenic epitopes (26, 52).

Given the vast diversity and biological uniqueness of bat species, extensive global collaborations have led to substantial progress in understanding bat-borne viruses (53), bat immune cells and molecules (54), and bat genomics/transcriptomics/immunomics (55). Annotated TR and IG loci from different bat species are gradually increasing, and the bat TCR CDR3 and BCR CDR3 repertoires are beginning to attract attention (27–29, 36). Although this study represents the first HTS sequencing and compositional analysis of the BCR CDR3 repertoire in spleen and intestine samples of *Hipposideros armiger* and *Rhinolophus pearsonii*, it is important to emphasize that the number and species of bats collected in the field were very limited (total of 10 samples), and we did not screen or control for age, sex, or viral carriage, precluding stratified analysis of detailed BCR CDR3 repertoire characteristics. Furthermore, substantial individual variation was observed in the number of CDR3 sequences from intestinal samples. Regarding control groups, we used peripheral blood BCR CDR3 repertoires from healthy volunteers and bone marrow BCR CDR3 repertoires from mice, which do not enable high-quality comparison with the bat spleen and intestinal BCR CDR3 repertoires, nor can we exclude potential effects of regional tissue-specific B cell differences. Nevertheless, this study addresses the current gap in HTS analysis of the bat IG light chain BCR CDR3 repertoire by successfully establishing, for the first time, a platform for library construction and HTS of BCR CDR3 from different bat species and tissue sites. We have preliminarily analyzed and characterized the general features of the BCR CDR3 repertoire (e.g., diversity, overlap, V gene and J gene usage bias), as well as its overall similarities and differences with human and mouse BCR CDR3 repertoires. Future studies with larger sample sizes and more refined sample classification are warranted to verify these differences. Overall, these findings provide new insights, comparative base-data, and technical approaches for further investigation of B-cell response efficacy and mechanisms in bats.

## Data Availability

The datasets presented in this study can be found in online repositories. The names of the repository/repositories and accession number(s) can be found below: https://www.ncbi.nlm.nih.gov/, PRJNA1277807, PRJNA921709, PRJNA578033, PRJEB33490, PRJEB43948, PRJNA1419000, PRJNA1417727.

## Glossary

BCR: B Cell Receptor
CDR3: Complementarity Determining Region 3
Cytb: Cytochrome B
Caspase-1: Cysteine-requiring Aspartate Protease 1
HTS: High-Throughput Sequencing
IGH: Immunoglobulin Heavy chain
IGL: Immunoglobulin Lambda chain
IGK: Immunoglobulin Kappa chain
IMGT: The International ImMunoGeneTics Database
IGHV: Immunoglobulin Heavy chain Variable gene
IGHJ: Immunoglobulin Heavy chain Jointing gene
IGHD: Immunoglobulin Heavy chain Diversity gene
IGHC: Immunoglobulin Heavy chain Constant Gene
IGLV: Immunoglobulin Lambda chain Variable gene
IGLJ: Immunoglobulin Lambda chain Jointing gene
IGKV: Immunoglobulin Kappa chain Variable gene
IGKJ: Immunoglobulin Kappa chain Jointing gene
IGLC: Immunoglobulin Lambda chain Constant Gene
IGKC: Immunoglobulin Kappa chain Constant Gene
LPS: Lipopolysaccharide
NLRP3: NOD-like receptor family, pyrin domain containing 3
N/P nucleotide: Non-templated(N)/Palindromic(P) nucleotide
RSS: Recombination Signal Sequence
PCR: Polymerase Chain Reaction
AA: Amino acid
NCBI: National Center for Biotechnology Information
IG: Immunoglobulin
ISGs: Interferon-stimulated genes
IFN: Interferon
TLR: Toll-like receptors
AIM2: Absent in melanoma 2
IL-1β: Interleukin - 1β
RIG-I: Retinoic Acid Induced Gene I
SEB: staphylococcal enterotoxin B.
Bat: Name*Hipposideros armiger*, IGH
Spleen: D2-S
Spleen: D3-S
*Hipposideros armiger*: IGH
Intestine: D2-I
Intestine: D3-I
*Rhinolophus*: IGH
Spleen: P1-S*(pearsonii)*
Spleen: P2-S*(pearsonii)*
*Rhinolophus*: IGH
Intestine: P1-I*(pearsonii)*
Intestine: P2-I*(pearsonii)*
*Rhinolophus pearsonii*: IGH
P2-IGH: 261244
P3-IGH: 741592
P4-IGH: 1050313
*Rhinolophus pearsonii*: IGL
P2-IGL: 16157961
P3-IGL: 11479657
P4-IGL: 13177606
*Rhinolophus pearsonii*: IGK
P2-IGK: 7194742
P3-IGK: 4123972
P4-IGK: 5426366
