# Supplementary material for: Annotation of bat IG H/L/K loci and analysis of the characteristics of bat BCR-CDR3 repertoires
Source: Front Immunol. 2026 May 20;17:1827051. doi: 10.3389/fimmu.2026.1827051 (PMC13229784; doi:10.3389/fimmu.2026.1827051)
Supplement: Supplementary file 1 [file DataSheet1.docx]

**Sup Tab 1.** Results of bat genotyping

| Sample | Cytb Sequences |  |
| --- | --- | --- |
| D1 | ATGACCAACATCCGCAAATCCCACCCACTATTCAAAATTATCAACGACTCATTCATTGATCTCCCCACCCCCTCAAGTATCTCATCCTGATGAAACTTCGGCTCCCTCTTAGGCATGTGCCTAGCAATCCAAATCCTGACAGGCCTATTCCTAGCAATACATTACACATCCGACACCGCCACCGCCTTTTACTCCGTAACCCATATCTGCCGAGACGTCAACTACGGCTGAATCCTACGATATCTCCACGCCAACGGAGCTTCCATATTCTTCATCTGCC  TGTTTCTACACGTAGGCCGAGGCATTTACTATGGCTCCTACACATACACAGAAACATGAAACATCGGAATTATTCTCCTCTTCACCGTCATAGCAACAGCATTTATAGGGTATGTCCTCCCATGAGGACAAATATCTTTCTGAGGCGCAACAGTCATCACCAACCTTCTCTCAGCCATCCCCTATATTGGAACCAACCTCGTAGAGTGGGTCTGAGGTGGCTTCTCCGTAGACAAAGCCACTCTCACTCGAT | *Hipposideros armige* |
| D2 | ATGACCAACATCCGCAAATCCCACCCACTATTCAAAATTATCAACGACTCATTCATTGATCTCCCCACCCCCTCAAGTATCTCATCCTGATGAAACTTCGGCTCCCTCTTAGGCATGTGCCTAGCAATCCAAATCCTGACAGGCCTATTCCTAGCAATACATTACACATCCGACACCGCCACCGCCTTTTACTCCGTAACCCATATCTGCCGAGACGTCAACTACGGCTGAATCCTACGATATCTCCACGCCAACGGAGCTTCCATATTCTTCATCTGCCTGTTTCTACACGTAGGCCGAGGCATTTACTATGGCTCCTACACATACACAGAAACATGAAACATCGGAATTATTCTCCTCTTCACCGTCATAGCAACAGCATTTATAGGGTATGTCCTCCCATGAGGACAAATATCTTTCTGAGGCGCAACAGTCATCACCAACCTTCTCTCAGCCATCCCCTATATTGGAACCAACCTCGTAGAGTGGGTCTGAGGTGGCTTCTCCGTAGACAAAGCCACTCTCACTCGATTCTTT | *Hipposideros armige* |
| D3 | ATGACCAACATCCGCAAATCCCACCCACTATTCAAAATTATCAACGACTCATTCATTGATCTCCCCACCCCCTCAAGTATCTCATCCTGATGAAACTTCGGCTCCCTCTTAGGCATGTGCCTAGCAATCCAAATCCTGACAGGCCTATTCCTAGCAATACATTACACATCCGACACCGCCACCGCCTTTTACTCCGTAACCCATATCTGCCGAGACGTCAACTACGGCTGAATCCTACGATATCTCCACGCCAACGGAGCTTCCATATTCTTCATCTGCCTGTTTCTACACGTAGGCCGAGGCATTTACTATGGCTCCTACACATACACAGAAACATGAAACATCGGAATTATTCTCCTCTTCACCGTCATAGCAACAGCATTTATAGGGTATGTCCTCCCATGAGGACAAATATCTTTCTGAGGCGCAACAGTCATCACCAACCTTCTCTCAGCCATCCCCTATATTGGAACCAACCTCGTAGAGTGGGTCT | *Hipposideros armige* |
| P1 | GTTGACTTACCAACCCCATCAAGTATCTCCTCCTGATGAAACTTCGGATECCTECTAGGAGTCTGCCTAGCCATACAAATCCTCACGGGTCTATTCCAGCTATACACTACACATCAGACACCGCCACGGCCTTCTACTCCGTAACCCATATTTGCCGAGACETCAACTACGGCTGAATTCTACGCACCTTCACGCCAACGGAGCCFCCATATTTTTCATCTGCCTATTCCTGCACGAGGACGGGGAATCTACTACGGATCCTACACATTCTCGGAAACATGAAACGTCGGAATCATCCTCTTATCGCCGTCATGGCTACAGCATTCATAGGCTACGTCCTCCCATGAGGCCAAATATCTTCTGAGGTGCAACGGTCATCACAAACCTTCFCTCGGCCATCCTTACATTGGAACTACCCTGGTAGAATGAGTCTGAGGGGGATTCTCGGTTGACAAAGCCACACTTACCCG | *Rhinolophus pearsonii* |
| P2 | GTTGACTTACCAACCCCATCAAGTATCTCCTCCTGATGAAACTTCGGATECCTECTAGGAGTCTGCCTAGCCATACAAATCCTCACGGGTCTATTCCAGCTATACACTACACATCAGACACCGCCACGGCCTTCTACTCCGTAACCCATATTTGCCGAGACETCAACTACGGCTGAATTCTACGCACCTTCACGCCAACGGAGCCFCCATATTTTTCATCTGCCTATTCCTGCACGAGGACGGGGAATCTACTACGGATCCTACACATTCTCGGAAACATGAAACGTCGGAATCATCCTCTTATCGCCGTCATGGCTACAGCATTCATAGGCTACGTCCTCCCATGAGGCCAAATATCTTCTGAGGTGCAACGGTCATCACAAACCTTCFCTCGGCCATCCTTACATTGGAACTACCCTGGTAGAATGAGTCTGAGGGGGATTCTCGGTTGACAAAGCCACACTTACCCG | *Rhinolophus pearsonii* |
| P3 | TAATGACCCAAACACCGGAAATCCATCCACTATTCAAAATTATCAACGACTCATTCGTTGACTTACCAACCCCATCAAGCATCTCCTCCTGATGAAACTTCGGATCCCTCCTGGGAATCTGCCTAGCCATACAAATCCTCACGGGTCTCTTCCTAGCTATACACTACACATCAGACACCGCCACGGCCTTCTACTCCGTAACCCATATTTGCCGAGACGTCAACTACGGCTGAATTCTACGCTACCTTCACGCCAACGGAGCCTCCATATTCTTCATCTGCCTATTCCTGCACGTAGGACGGGGAATCTACTACGGATCCTACACATTCTCGGAAACATGAAACGTTGGAATCATCCTCCTATTCGCCGTCATGGCTACAGCATTCATAGGCTACGTCCTCCCATGAGGCCAAATGTCTTTCTGAGGTGCAACGGTCATCACAAACCTTCTCTCGGCCATCCCCTACATTGGGACTACCCTGGTAGAATGGGTCTGAGGAGGATTCTCAGTTGACAAAGCCACACTTACCCGATTCTTCGCCTC | *Rhinolophus pearsonii* |
| P4 | ACCGGAAATCCACCCACTATTCAAAATTATCAACGACTCATTCGTTGACTTACCAACCCCATCAAGCATCTCCTCCTGATGAAACTTCGGATCCCTCCTGGGAATCTGCCTAGCCATACAAATCCTCACGGGTCTCTTCCTAGCTATACACTACACATCAGACACCGCCACGGCCTTCTACTCCGTAACCCATATTTGCCGAGACGTCAACTACGGCTGAATTCTACGCTACCTTCACGCCAACGGAGCCTCCATATTCTTCATCTGCCTATTCCTGCACGTAGGACGGGGAATCTACTACGGATCCTACACATTCTCGGAAACATGAAACGTTGGAATCATCCTCCTATTCGCCGTCATGGCTACAGCATTCATAGGCTACGTCCTCCCATGAGGCCAAATGTCTTTCTGAGGTGCAACGGTCATCACAAACCTTCTCTCGGCCATCCCCTACATTGGGACTACCCTGGTAGAATGGGTCTGAGGAGGATTCTCAGTTGACAAAGCCACACTTACCCGATTCTTCGCCTCCCC | *Rhinolophus pearsonii* |
| P5 | ACCGGAAATCAATCCACTATTCAAAATTATCAACGACTCATTCGTTGACTTACCAACCCCATCAAGCATCTCCTCCTGATGAAACTTCGGATCCCTCCTGGGAATCTGCCTAGCCATACAAATCCTCACGGGTCTCTTCCTAGCTATACACTACACATCAGACACCGCCACGGCCTTCTACTCCGTAACCCATATTTGCCGAGACGTCAACTACGGCTGAATTCTACGCTACCTTCACGCCAACGGAGCCTCCATATTCTTCATCTGCCTATTCCTGCACGTAGGACGGGGAATCTACTACGGATCCTACACATTCTCGGAAACATGAAACGTTGGAATCATCCTCCTATTCGCCGTCATGGCTACAGCATTCATAGGCTACGTCCTCCCATGAGGCCAAATGTCTTTCTGAGGTGCAACGGTCATCACAAACCTTCTCTCGGCCATCCCCTACATTGGGACTACCCTGGTAGAATGGGTCTGAGGAGGATTCTCAGTTGACAAAGCCACACTTACCCGATTCTTCGCCTC | *Rhinolophus pearsonii* |
| P6 | CCTCGGAAATCCATCCACTATTCAAAATTATCAACGACTCATTCGTTGACTTACCAACCCCATCAAGCATCTCCTCCTGATGAAACTTCGGATCCCTCCTGGGAATCTGCCTAGCCATACAAATCCTCACGGGTCTCTTCCTAGCTATACACTACACATCAGACACCGCCACGGCCTTCTACTCCGTAACCCATATTTGCCGAGACGTCAACTACGGCTGAATTCTACGCTACCTTCACGCCAACGGAGCCTCCATATTCTTCATCTGCCTATTCCTGCACGTAGGACGGGGAATCTACTACGGATCCTACACATTCTCGGAAACATGAAACGTTGGAATCATCCTCCTATTCGCCGTCATGGCTACAGCATTCATAGGCTACGTCCTCCCATGAGGCCAAATGTCTTTCTGAGGTGCAACGGTCATCACAAACCTTCTCTCGGCCATCCCCTACATTGGGACTACCCTGGTAGAATGGGTCTGAGGAGGATTCTCAGTTGACAAAGCCACACTTACCCGATTCTTCGCCTCCCCC | *Rhinolophus pearsonii* |
| F1 | ATGACCAACATTCGTAAATCCCACCCATTATTTAAGATCATCAACGACTCGTTCATCGACCTACCAGCCCCATCAAGCATCTCCTCCTGATGAAACTTCGGGTCCCTACTAGGAGTCTGCCTAGCCGTACAAATCCTCACAGGCCTATTCCTGGCCATACACTACACATCAGACACCGCCACTGCCTTCTACTCCGTAACCCATATCTGCCGAGACGTCAATTACGGCTGAGTTCTACGCTACCTCCACGCCAACGGAGCCTCCATATTCTTCATCTGCCTATTTCTGCATGTAGGACGGGGAATCTACTATGGCTCCTATACATTCTCCGAGACATGAAATGTCGGAATTATTCTCCTCTTCGCCGTTATAGCCACAGCATTCATAGGCTACGTACTTCCGTGAGGCCAAATATCCTTCTGAGGAGCAACGGTCATTACAAACCTCCTCTCAGCCATTCCATACGTCGGAACAACTCTAGTAGAATGAGTCTGAGGGGGATTCTCAGTAGACAAAGCCACACTCACCCGATTCTTCGCCCTA | *Rhinolophus pusillus* |

**Sup Tab 2.** Human BCRCDR3 HTS Data Sheet

| Name | Number | Sample | Productive | Clonotype |
| --- | --- | --- | --- | --- |
| IGH | PRJNA1417727 | H1-IGH | 413,204 | 28955 |
|  | PRJNA1417727 | H2-IGH | 329,736 | 26111 |
|  | PRJNA1417727 | H3-IGH | 275,755 | 22595 |
|  | ERR3445161 | H4-IGH | 992,881 | 159621 |
| IGL | SRR23010845 | H1-IGL | 2,807,592 | 3396 |
|  | SRR23010851 | H2-IGL | 2,424,988 | 441 |
|  | SRR23010854 | H3-IGL | 2,763,660 | 3999 |
|  | SRR10298045 | H4-IGL | 9,711,033 | 39587 |
| IGK | SRR23010849 | H1-IGK | 1,745,195 | 857 |
|  | SRR23010852 | H2-IGK | 1,021,313 | 423 |
|  | SRR23010859 | H3-IGK | 1,313,526 | 407 |
|  | SRR10298046 | H4-IGK | 6,815,102 | 52436 |

**Sup Tab 3.** Mouse BCRCDR3 HTS Data Sheet

| Name | Number | Sample | Productive | Clonotype |
| --- | --- | --- | --- | --- |
| IGH | PRJNA1419000 | M1-IGH | 2,724,205 | 374307 |
|  | PRJNA1419000 | M2-IGH | 3,133,607 | 543367 |
|  | PRJNA1419000 | M3-IGH | 1,528,623 | 194248 |
|  | ERR5556766 | M4-IGH | 892,186 | 50084 |
| IGL | SRR13344578 | M1-IGL | 23,849 | 53 |
|  | SRR13344580 | M2-IGL | 54,181 | 87 |
|  | SRR13344582 | M3-IGL | 35,128 | 42 |
|  | SRR13344584 | M4-IGL | 56,452 | 44 |
| IGK | SRR15183791 | M1-IGK | 13,003 | 582 |
|  | SRR15183790 | M2-IGK | 562,552 | 1779 |
|  | SRR15183794 | M3-IGK | 9,116 | 366 |
|  | SRR15183793 | M4-IGK | 533,957 | 1657 |

**Sup Tab 4.** Statistics of IGHV gene families in *Antrozous pallidus* and *Rhinolophus ferrumequinum*

| IGHV subgroup | Antrozous pallidus | | Rhinolophus ferrumequinum | |
| --- | --- | --- | --- | --- |
|  | direction | total | direction | total |
| *IGHV1* | forward/reverse | 14 | forward | 14 |
| *IGHV2* |  | 0 | forward | 6 |
| *IGHV3* | forward/reverse | 39 | forward | 13 |
| *IGHV4* | forward/reverse | 22 | forward | 5 |
| *IGHV5* |  | 0 |  | 0 |
| *IGHV6* |  | 0 |  | 0 |
| *IGHV7* |  | 0 |  | 0 |
| *IGHV8* |  | 0 |  | 0 |
| *IGHV9* |  | 0 |  | 0 |
| *IGHV10* |  | 0 |  | 0 |
| *IGHV11* | forward | 5 | forward | 2 |
| *IGHV(I)* |  | 0 |  | 0 |
| *IGHV(II)* |  | 0 |  | 0 |
| *IGHV(III)* | reverse | 1 | forward | 1 |
| Functional | forward/reverse | 65 | forward | 35 |
| ORF | forward | 3 | forward | 1 |
| Pseudogene | forward/reverse | 13 | forward | 5 |
| Total | forward/reverse | 81 | forward | 41 |

**Sup Tab 5.** Statistics of IGLV *Antrozous pallidus* and *Rhinolophus ferrumequinum*

| IGLV subgroup | Antrozous pallidus | | Rhinolophus ferrumequinum | |
| --- | --- | --- | --- | --- |
|  | direction | total | direction | total |
| *IGLV1* | forward | 9 | forward | 10 |
| *IGLV2* | forward | 5 | forward | 3 |
| *IGLV3* | forward | 5 | forward | 12 |
| *IGLV4* | forward | 2 | forward | 4 |
| *IGLV5* | forward | 14 | forward | 23 |
| *IGLV6* | forward | 1 | forward | 1 |
| *IGLV7* | forward | 5 | forward | 12 |
| *IGLV8* | forward | 9 | forward | 5 |
| *IGLV9* |  | 0 | forward | 2 |
| *IGLV10* |  | 0 | forward | 1 |
| *IGLV11* | forward | 2 |  | 0 |
| *IGLV13* | forward | 3 | forward | 1 |
| *IGKV(I)* |  | 0 |  | 0 |
| *IGLV(II)* |  | 0 |  | 0 |
| *IGLV(III)* | forward | 1 |  | 0 |
| Functional | forward | 52 | forward | 52 |
| ORF | forward | 0 | forward | 3 |
| Pseudogene | forward | 4 | forward | 19 |
| Total | forward | 56 | forward | 74 |

**Sup Tab 6.** Statistics of IGKV *Rhinolophus ferrumequinum*

| IGKV subgroup | Rhinolophus ferrumequinum | |
| --- | --- | --- |
|  | direction | total |
| *IGKV1* | forward | 15 |
| *IGKV2* |  | 0 |
| *IGKV3* | forward | 1 |
| *IGKV4* |  | 0 |
| *IGKV5* |  | 0 |
| *IGKV6* |  | 0 |
| *IGKV7* |  | 0 |
| *IGKV8* | forward | 1 |
| *IGKV9* |  | 0 |
| *IGKV10* |  | 0 |
| *IGKV11* |  | 0 |
| *IGKV(I)* |  | 0 |
| *IGKV(II)* |  | 0 |
| *IGKV(III)* |  | 0 |
| Functional | forward | 17 |
| ORF |  | 0 |
| Pseudogene |  | 0 |
| Total | forward | 17 |

**Sup Tab 7.** *Antrozous pallidus* IGHV pseudogene statistics

| IGHV | Antrozous pallidus | |
| --- | --- | --- |
|  | Functionality | Stop codon |
| IGHV1-7 | P |  |
| IGHV1-9 | P |  |
| IGHV3-12 | P |  |
| IGHV3-16 | P |  |
| IGHV3-25 | P |  |
| IGHV11-1 | P |  |
| IGHV3-18 | P |  |
| IGHV1-13 | P |  |
| IGHV4-20 | P |  |
| IGHV4-21 | P |  |
| IGHV3-39 | P |  |
| IGHV11-5 | P |  |
| IGHV(III)-1 | P |  |

**Sup Tab 8.**  *Antrozous pallidus /Rhinolophus ferrumequinum* IGLV pseudogene statistics

| IGLV Functionality | Rhinolophus ferrumequinum | Antrozous pallidus |
| --- | --- | --- |
| P | IGLV5-3 | IGLV1-6 |
| P | IGLV7-1 | IGLV1-7 |
| P | IGLV9-1 | IGLV4-2 |
| P | IGLV5-10 | IGLV(III)-1 |
| P | IGLV5-13 |  |
| P | IGLV5-14 |  |
| P | IGLV1-4 |  |
| P | IGLV5-16 |  |
| P | IGLV5-17 |  |
| P | IGLV5-20 |  |
| P | IGLV13-1 |  |
| P | IGLV5-23 |  |
| P | IGLV1-8 |  |
| P | IGLV4-1 |  |
| P | IGLV4-3 |  |
| P | IGLV3-7 |  |
| P | IGLV4-4 |  |
| P | IGLV3-11 |  |
| P | IGLV3-12 |  |

**Sup Tab 9.** Statistics on the number of IGH genes in bats and other species

| Species | IGHV | IGHD | IGHJ | IGHC |
| --- | --- | --- | --- | --- |
| *Bos taurus* | 50 | 23 | 12 | 12 |
| *Canis lupus familiaris* | 89 | 6 | 6 | 8 |
| *Danio rerio* | 41 | 7 | 7 | 3 |
| *Equus caballus* | 104 | 44 | 9 | 11 |
| *Gallus gallus* | 104 | 4 | 1 | 3 |
| *Gorilla gorilla gorilla* | 157 | 32 | 9 | 13 |
| *Homo sapiens* | 205 | 37 | 9 | 13 |
| *Macaca fascicularis* | 149 | 40 | 8 | 7 |
| *Macaca mulatta* | 293 | 45 | 7 | 8 |
| *Mus musculus* | 356 | 34 | 4 | 9 |
| *Oncorhynchus mykiss* | 224 | 37 | 21 | 5 |
| *Ornithorhynchus anatinus* | 69 | 3 | 11 | 8 |
| *Oryctolagus cuniculus* | 67 | 11 | 6 | 19 |
| *Rattus norvegicus* | 402 | 35 | 4 | 8 |
| *Salmo salar* | 303 | 28 | 21 | 12 |
| *Vicugna pacos* | 88 | 8 | 7 | 8 |
| *Rhinolophus ferrumequinum* | 41 | 4 | 6 | 4 |
| *Phyllostomus discolor* | 81 | 16 | 7 | 4 |
| *Pipistrellus pipistrellus* | 57 | 7 | 6 | 4 |
| *Rousettus aegyptiacu* | 66 | 8 | 9 | 4 |
| *Antrozous pallidus* | 81 | 16 | 6 | 5 |

**Sup Tab 10.** Statistics on the number of IGL genes in bats and other species

| **Species** | **IGLV** | **IGLJ** | **IGLC** |
| --- | --- | --- | --- |
| *Bos taurus* | 74 | 9 | 9 |
| *Canis lupus familiaris* | 261 | 9 | 9 |
| *Homo sapiens* | 83 | 7 | 7 |
| *Mus musculus* | 8 | 4 | 4 |
| *Gallus gallus* | 34 | 1 | 1 |
| *Felis catus* | 94 | 12 | 11 |
| *Capra hircus* | 73 | 2 | 2 |
| *Ovis aries* | 127 | 2 | 2 |
| *Macaca mulatta* | 149 | 8 | 8 |
| *Gorilla gorilla gorilla* | 86 | 7 | 7 |
| *Lemur catta* | 166 | 10 | 10 |
| *Pongo abelii* | 105 | 7 | 7 |
| *Oryctolagus cuniculus* | 43 | 4 | 6 |
| *Rattus norvegicus* | 8 | 4 | 4 |
| *Sus scrofa* | 21 | 3 | 2 |
| *[Rhinolophus ferrumequinum](https://www.ncbi.nlm.nih.gov/datasets/taxonomy/59479)* | 74 | 9 | 6 |
| *Antrozous pallidus* | 56 | 9 | 9 |

**Sup Tab 11.** Statistics on the number of IGK genes in bats and other species

| **Species** | **IGKV** | **IGKJ** | **IGKC** |
| --- | --- | --- | --- |
| *Homo sapiens* | 109 | 5 | 1 |
| *Mus musculus* | 222 | 5 | 1 |
| *Sus scrofa* | 17 | 5 | 1 |
| *Canis lupus familiaris* | 27 | 5 | 1 |
| *Capra hircus* | 21 | 4 | 1 |
| *Ovis aries* | 18 | 4 | 1 |
| *Bos taurus* | 25 | 5 | 1 |
| *Felis catus* | 18 | 5 | 1 |
| *Equus caballus* | 66 | 5 | 1 |
| *Camelus dromedarius* | 32 | 5 | 1 |
| *Macaca mulatta* | 110 | 5 | 1 |
| *Lemur catta* | 17 | 5 | 1 |
| *Gorilla gorilla gorilla* | 44 | 5 | 1 |
| *Pongo abelii* | 77 | 5 | 1 |
| *Pongo pygmaeus* | 73 | 5 | 1 |
| *Mustela putorius furo* | 87 | 5 | 1 |
| *Rattus norvegicus* | 165 | 7 | 1 |
| *[Rhinolophus ferrumequinum](https://www.ncbi.nlm.nih.gov/datasets/taxonomy/59479)* | 17 | 4 | 1 |
