# Supplementary material for: Annotation of bat IG H/L/K loci and analysis of the characteristics of bat BCR-CDR3 repertoires
Source: Front Immunol. 2026 May 20;17:1827051. doi: 10.3389/fimmu.2026.1827051 (PMC13229784; doi:10.3389/fimmu.2026.1827051)
Supplement: Supplementary file 2 [file DataSheet2.docx]

**A**

**B**

**Sup Fig 1.** Bat IGLC/IGKC sequence comparison

**A.** Bat IGLC sequence comparison

**B.** Bat IGKC sequence comparison

**
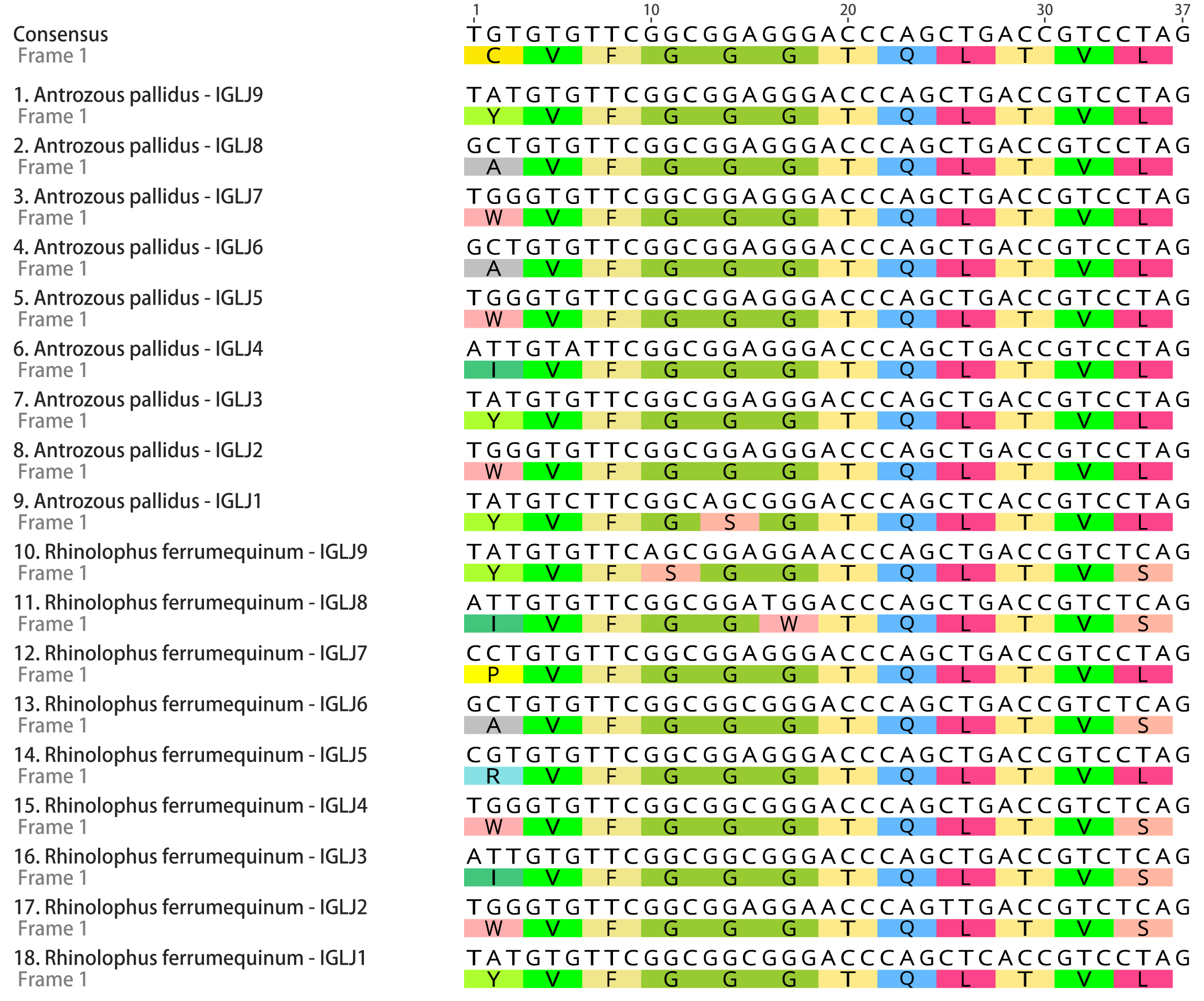

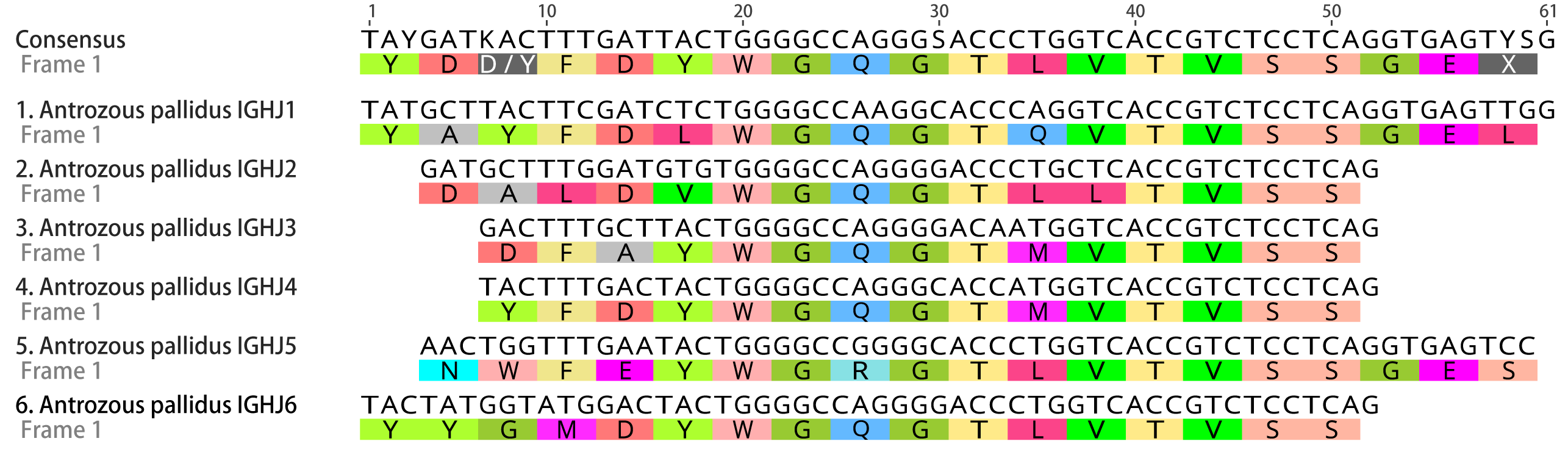

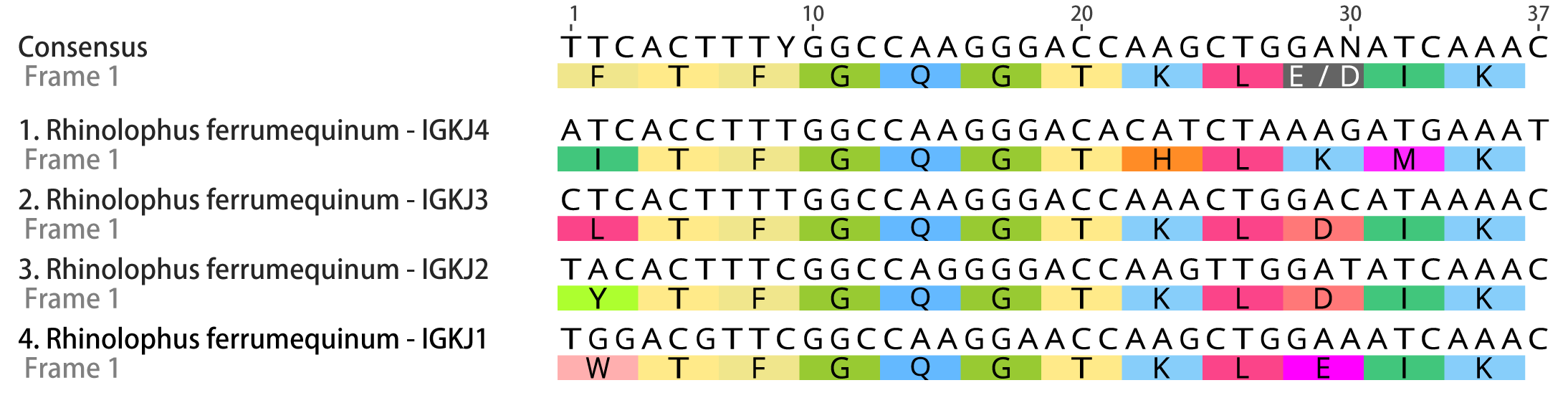
**

**Sup Fig 2.** Bat IGHJ/IGLJ/IGKJ amino acid nomenclature, comparative composition analysis

**A**

**B**

**C**

**
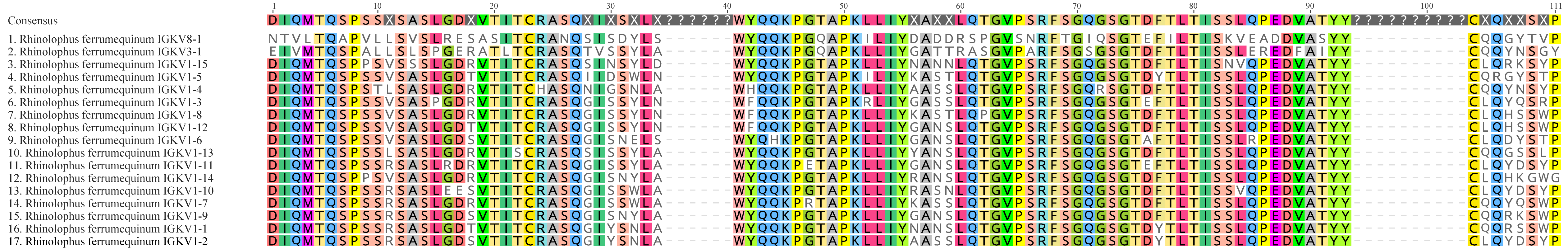
**

**D**

**Sup Fig 3.** Amino acid comparison of the IGHV/IGLV gene of *Antrozous pallidus* and the IGLV/IGKV gene of the *Rhinolophus ferrumequinu*m

**A.** Amino acid comparison of the IGHV gene of the *Antrozous pallidus*

**B.** Amino acid comparison of the IGLV gene of the *Antrozous pallidus*

**C.** Amino acid comparison of the IGLV gene of the *Rhinolophus ferrumequinum*

**D.** Amino acid comparison of the IGKV gene of the *Rhinolophus ferrumequinum*
