# Supplementary material for: Annotation of bat IG H/L/K loci and analysis of the characteristics of bat BCR-CDR3 repertoires
Source: Front Immunol. 2026 May 20;17:1827051. doi: 10.3389/fimmu.2026.1827051 (PMC13229784; doi:10.3389/fimmu.2026.1827051)
Supplement: Supplementary file 3 [file DataSheet3.pdf]

A

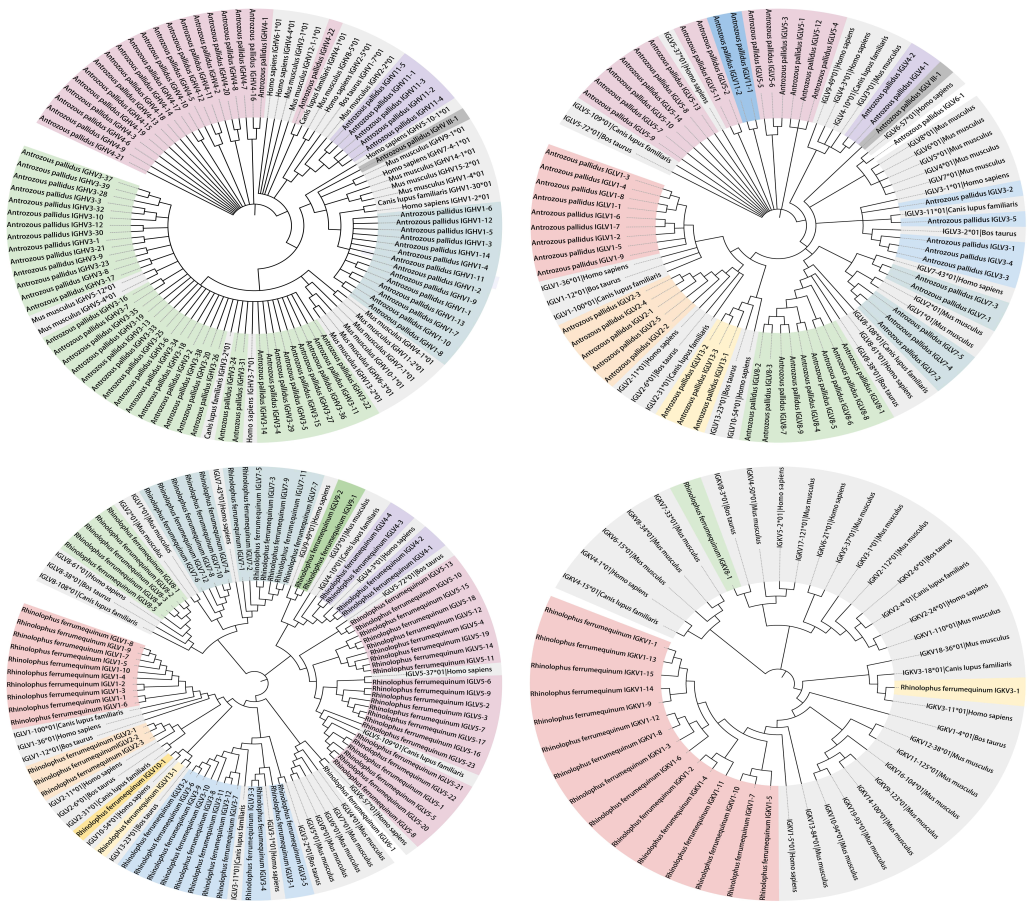

B

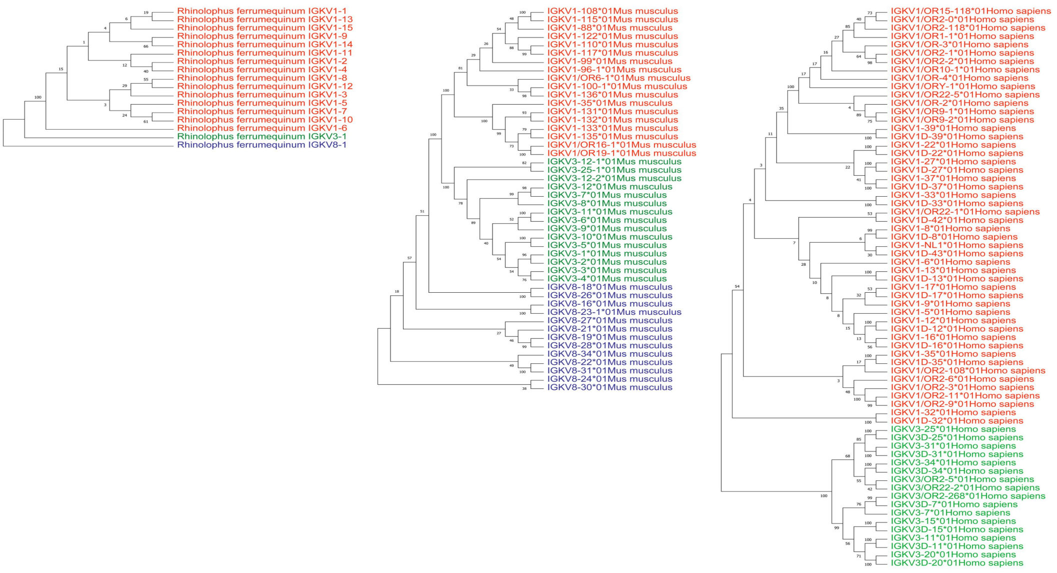

Sup Fig 4. Evolutionary tree analysis of IGHV/IGLV in the *Antrozous pallidus* and IGLV/IGKV in the *Rhinolophus ferrumequinum*

A.Evolutionary tree analysis of IGHV/IGLV in the *Antrozous pallidus* and IGLV/IGKV in the *Rhinolophus ferrumequinum*

B. Comparison of evolutionary analyses of IGKV (families 1,3,8) in the *Rhinolophus ferrumequinum*, human and mouse
