# Supplementary material for: Annotation of bat IG H/L/K loci and analysis of the characteristics of bat BCR-CDR3 repertoires
Source: Front Immunol. 2026 May 20;17:1827051. doi: 10.3389/fimmu.2026.1827051 (PMC13229784; doi:10.3389/fimmu.2026.1827051)
Supplement: Supplementary file 4 [file DataSheet4.docx]

**Sup Fig 5.** Bat, human, and mouse V/J RSS comparison

**A.** Bat, human, and mouse IGH RSS sequences

**B.** Bat, human, and mouse IGL RSS sequences; **C.** Bat, human, and mouse IGK RSS sequences


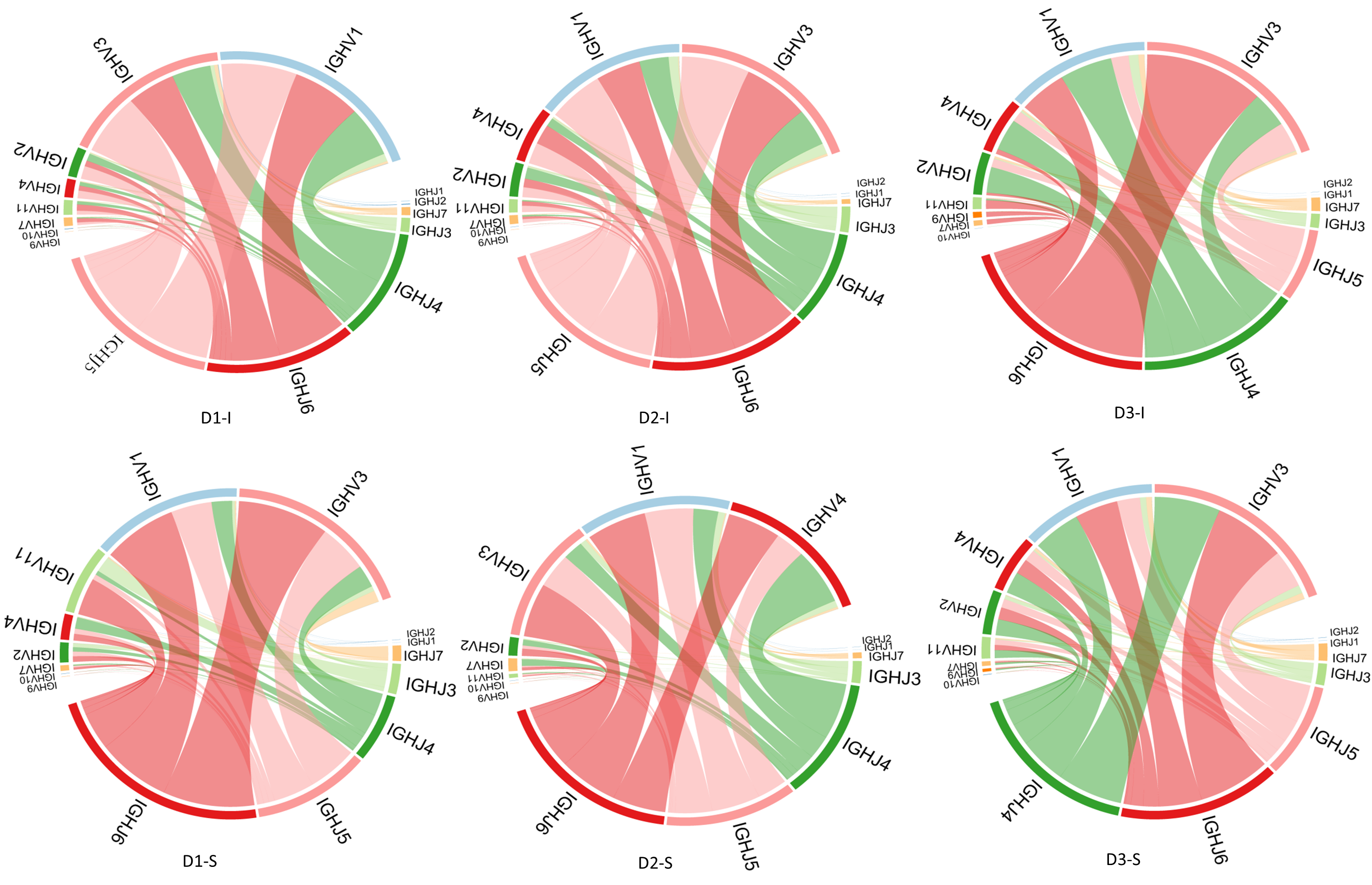


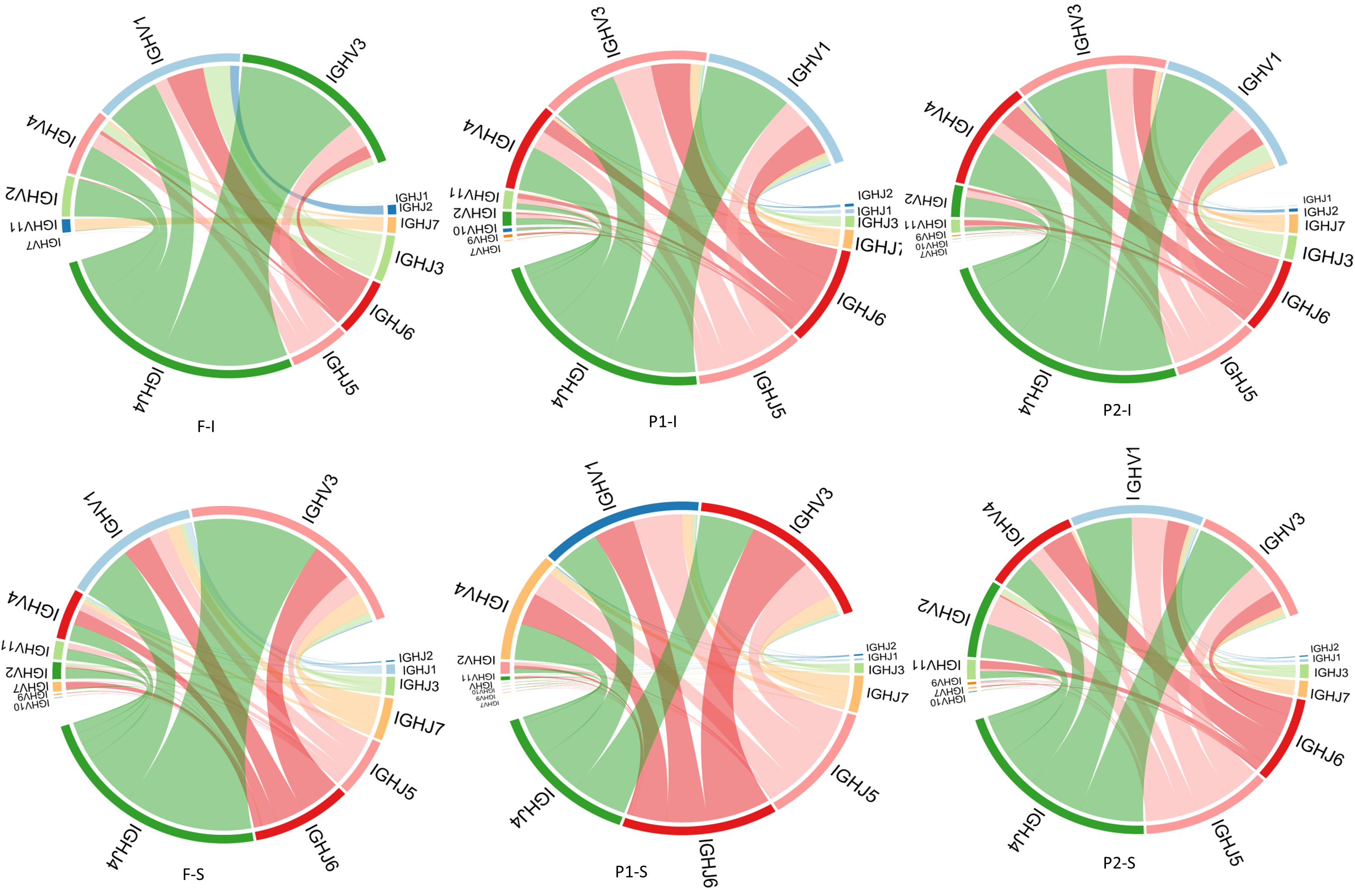
**Sup Fig 6.** V/J pairing of individual samples of spleen and intestinal IGH of *Hipposideros* and *Rhinolophus* bats

**
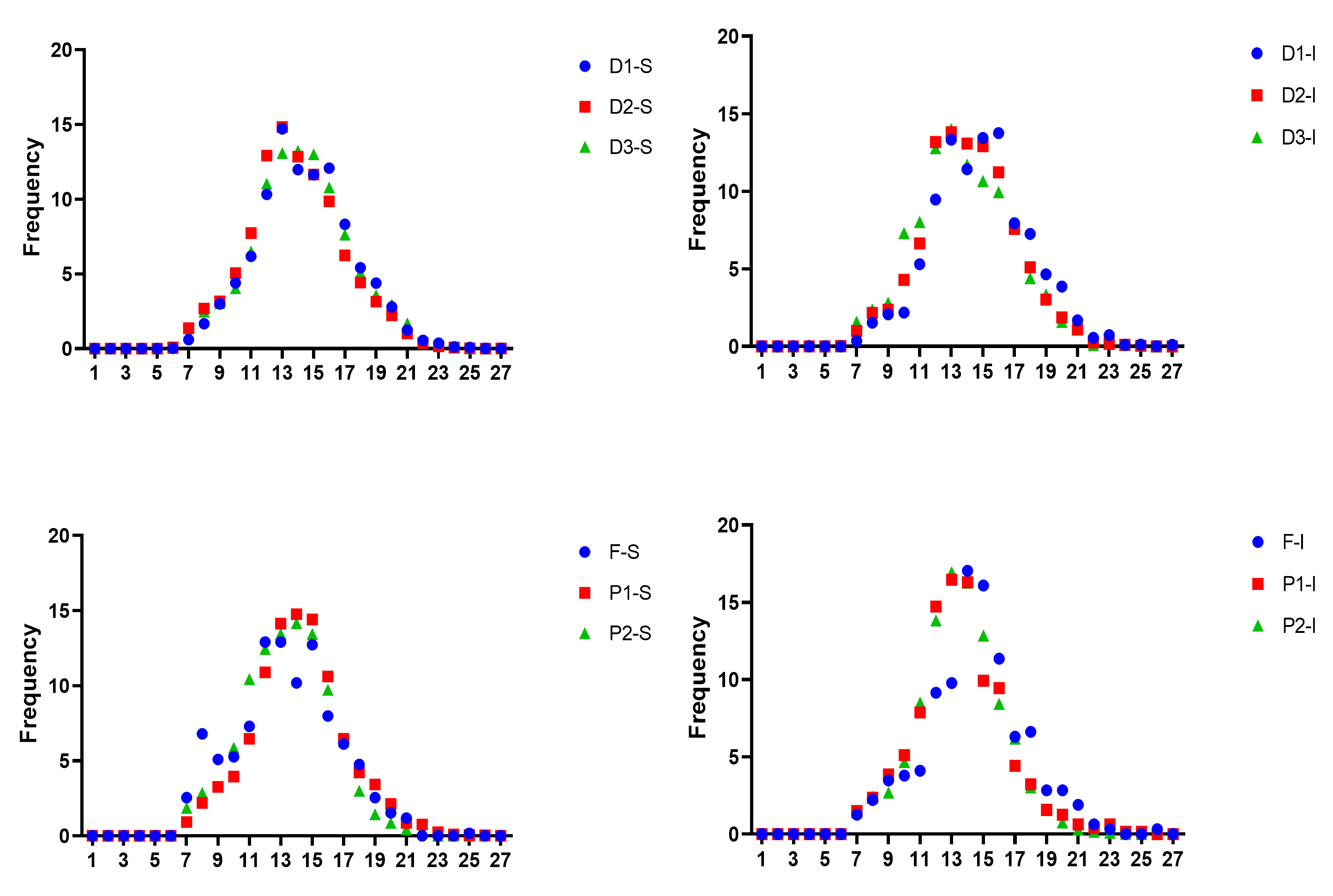
Sup Fig 7.** CDR3 length distribution of individual samples from spleen and intestinal IGH of *Hipposideros* and *Rhinolophus* bats

**
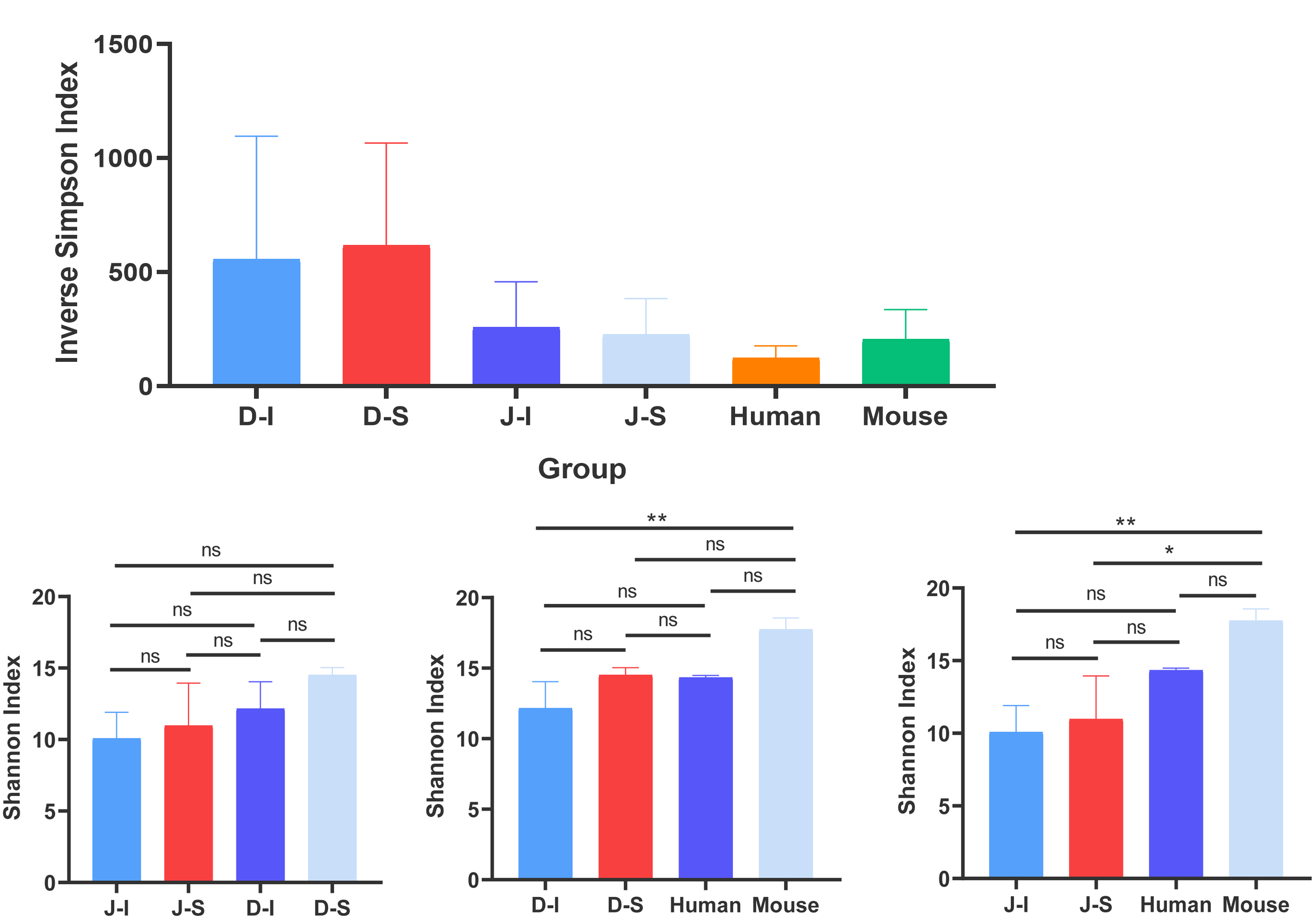
**

**Sup Fig 8.** Diversity analysis of bat intestinal and spleen, human, and mouse IGHomic libraries（Kruskal-Wallis H Test; one-way analysis of variance, P <0.05）

**
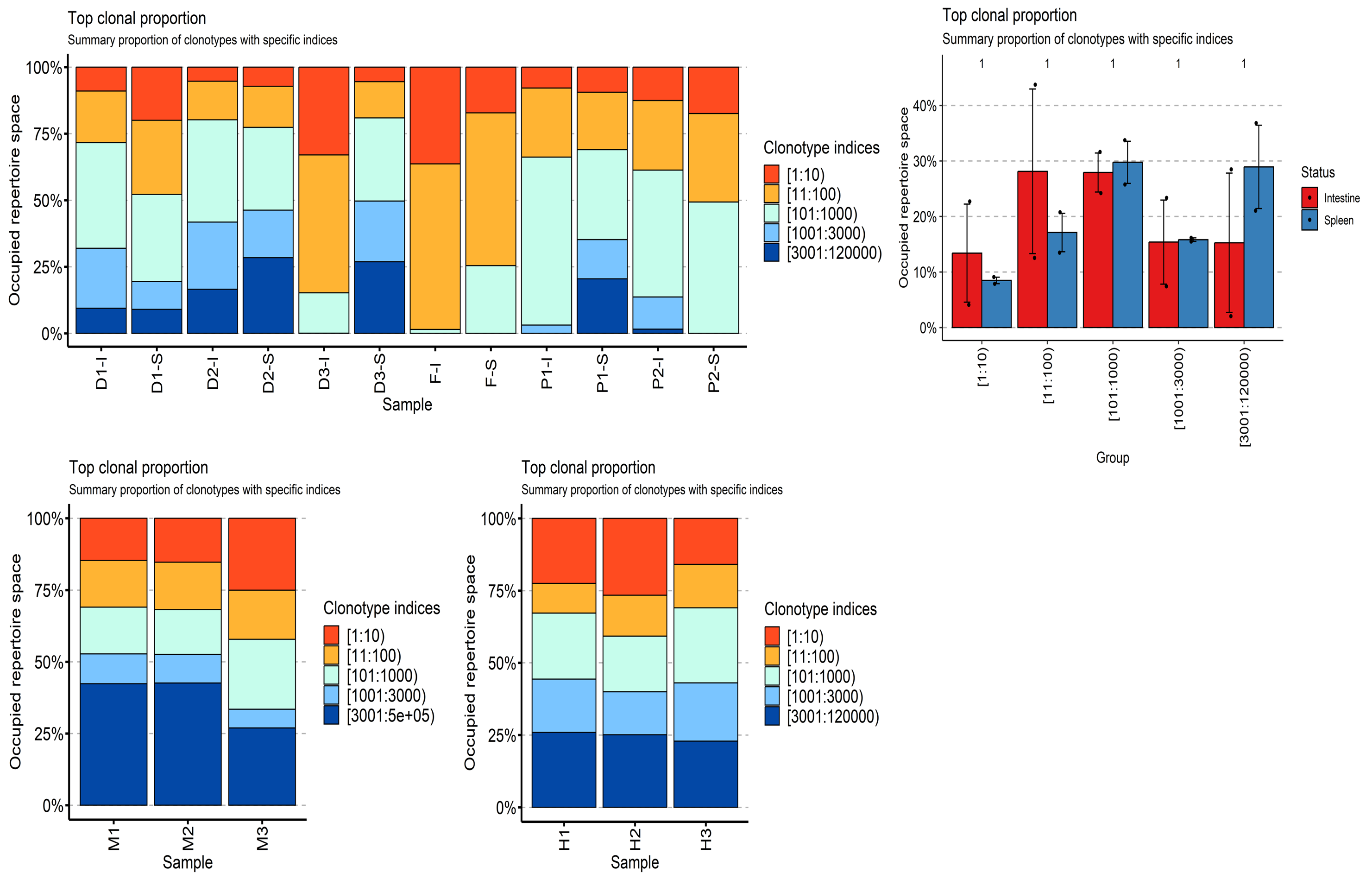
**

**Sup Fig 9.** Frequency distribution of high-frequency clones in bat intestine and spleen, human, and mouse（Kruskal-Wallis H Test,P <0.05）

**
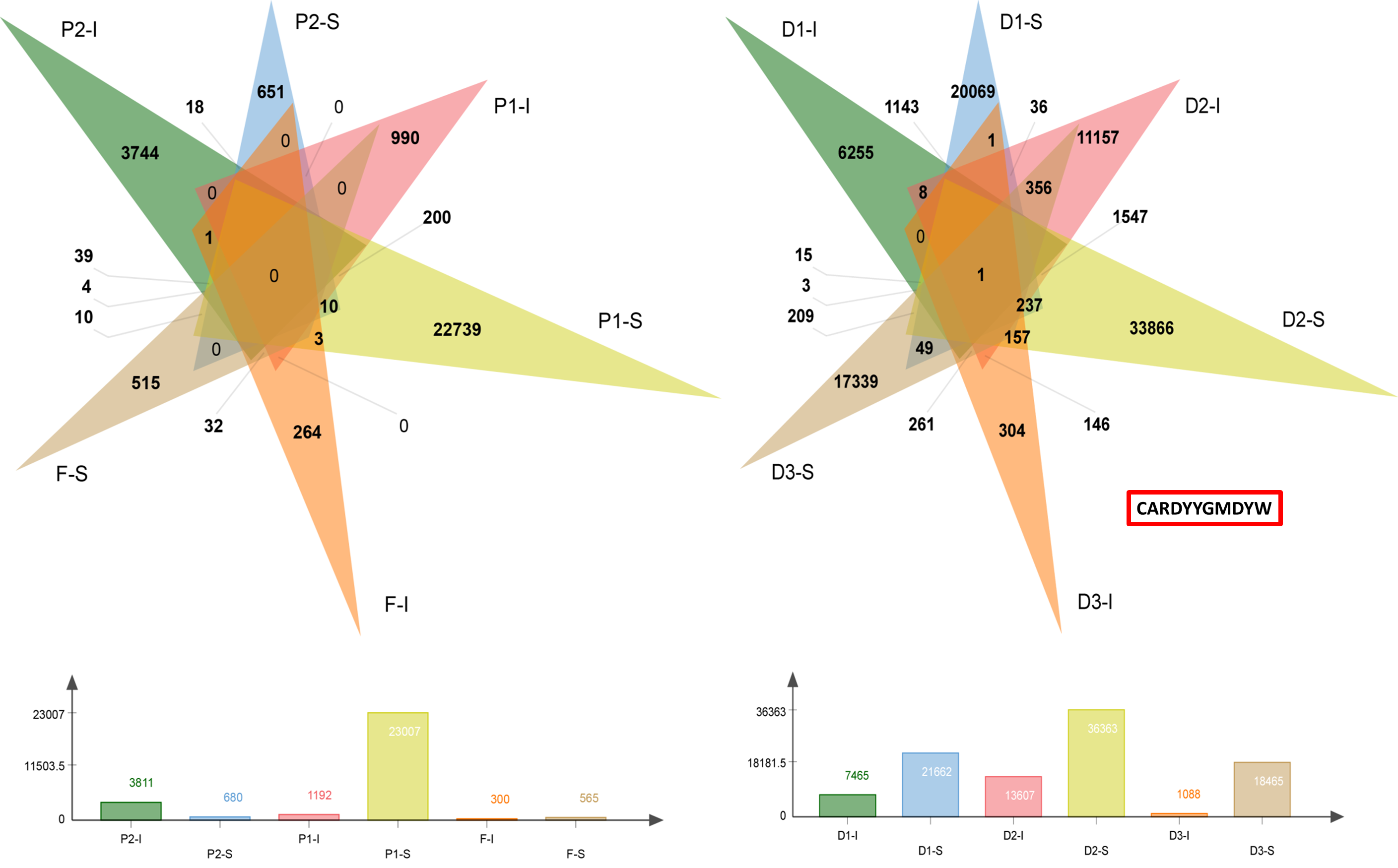
**

**Sup Fig 10.** Overlapping analysis of all samples of spleen and intestinal IGH of *Hipposideros* and *Rhinolophus* bats

**
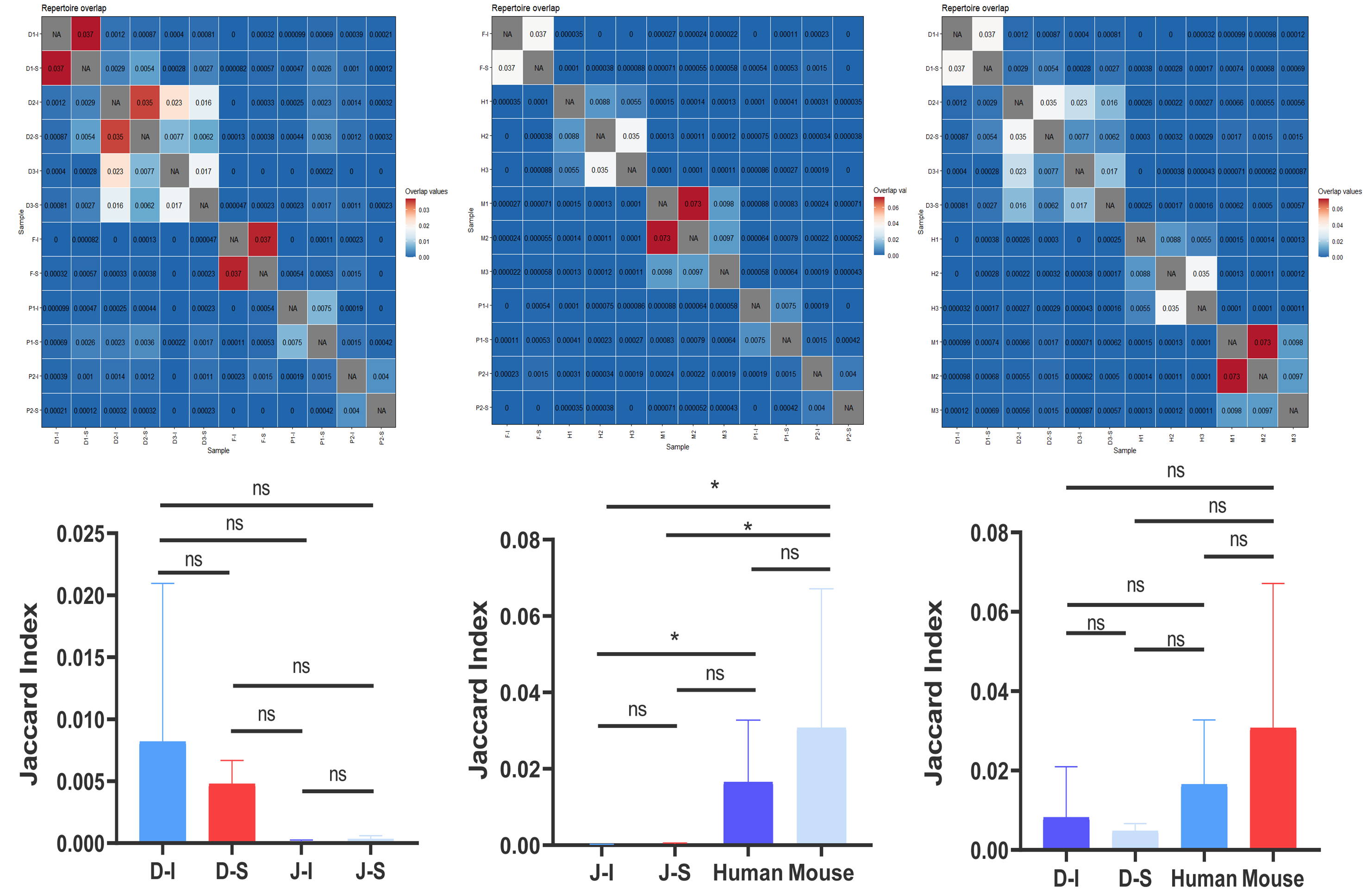
**

**Sup Fig 11.** Analysis of CDR3 overlap indices in bat intestine and spleen, human, mouse IGH（Kruskal-Wallis H Test,P <0.05）

**
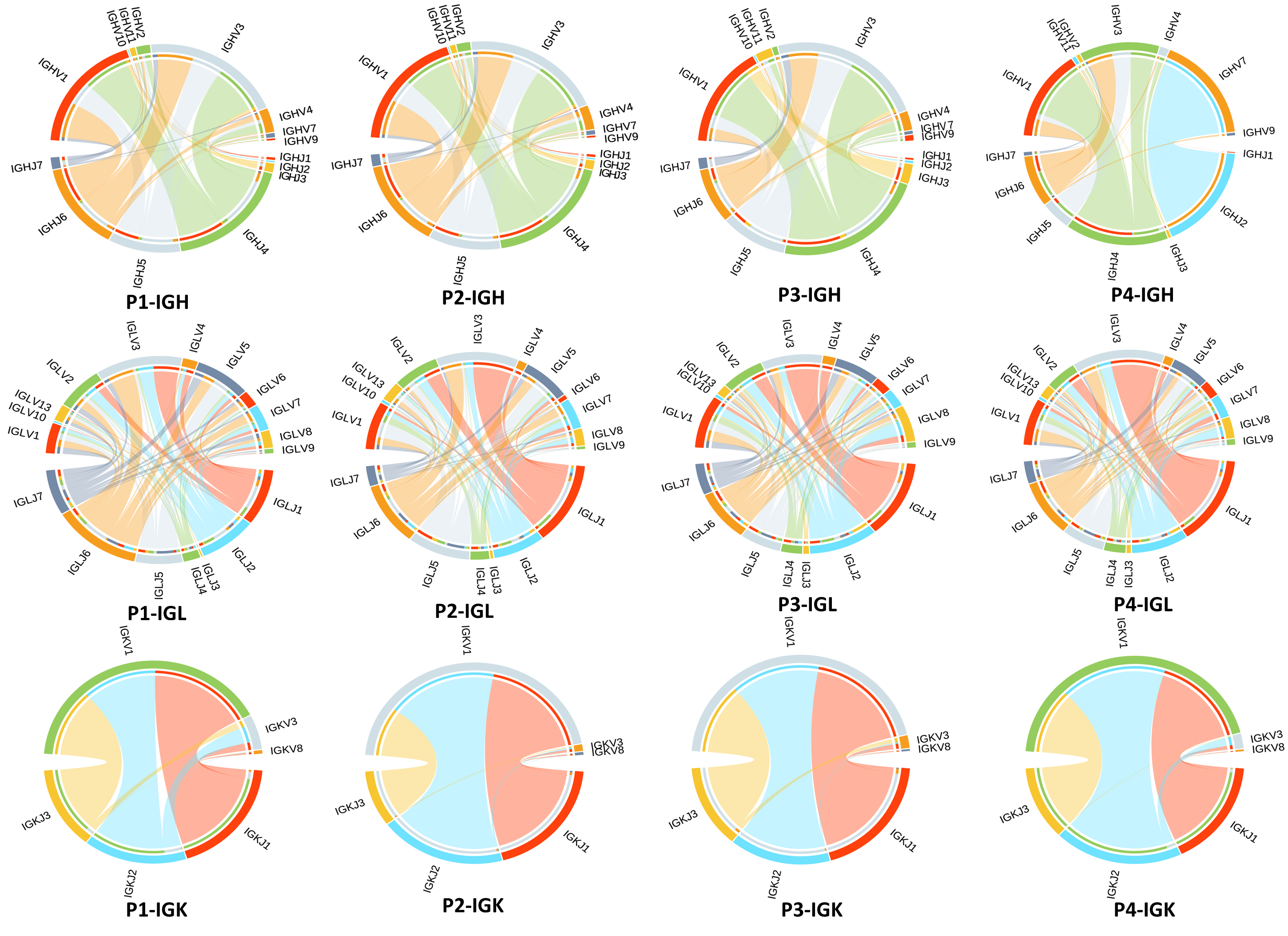
**

**Sup Fig 12.** V-J pairwise analysis of individual samples of bats IGH/IGL/IGK

**
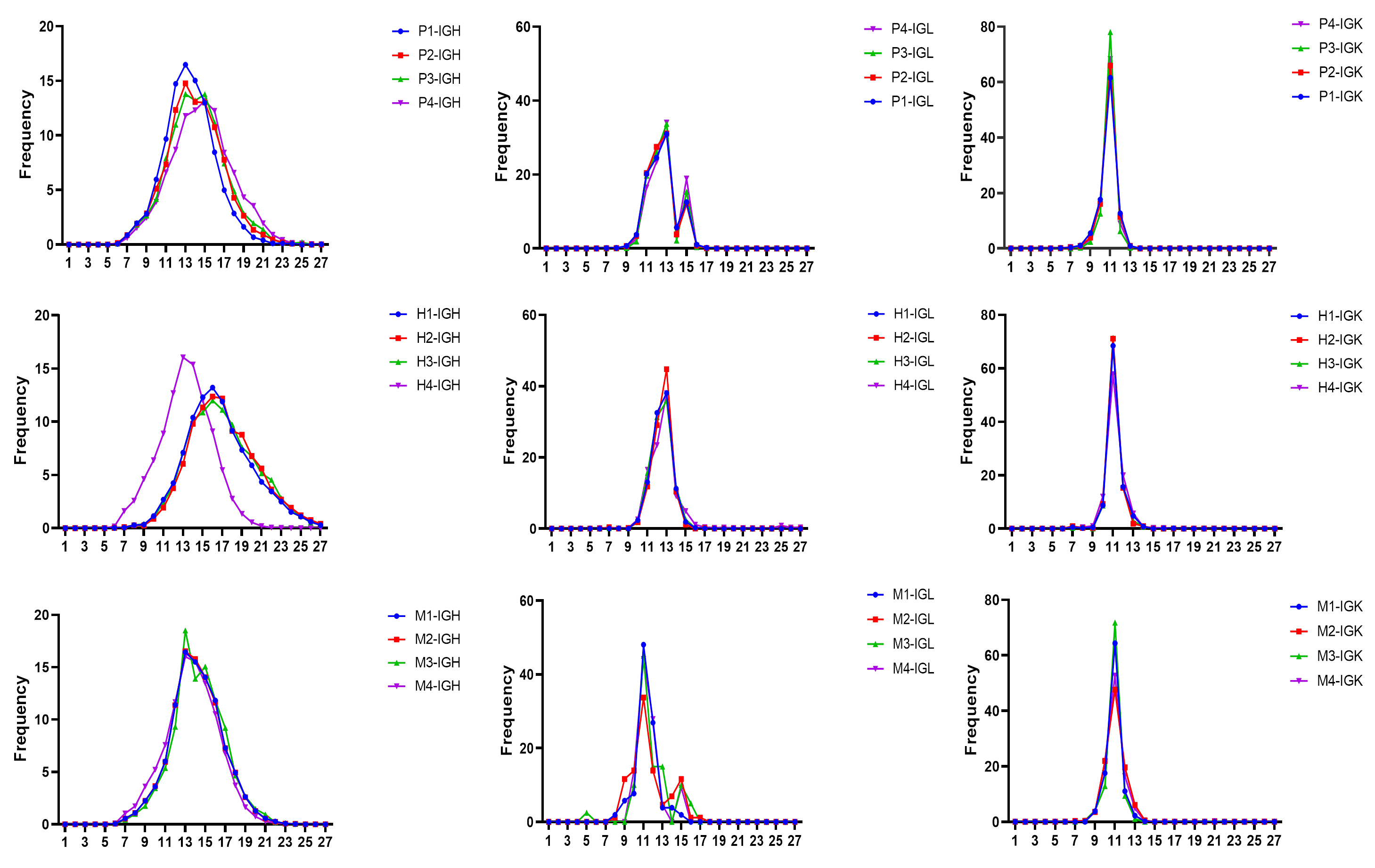
**

**Sup Fig 13.** CDR3 length distribution of individual samples from bat, human and mouse IGH/IGL/IGK

**
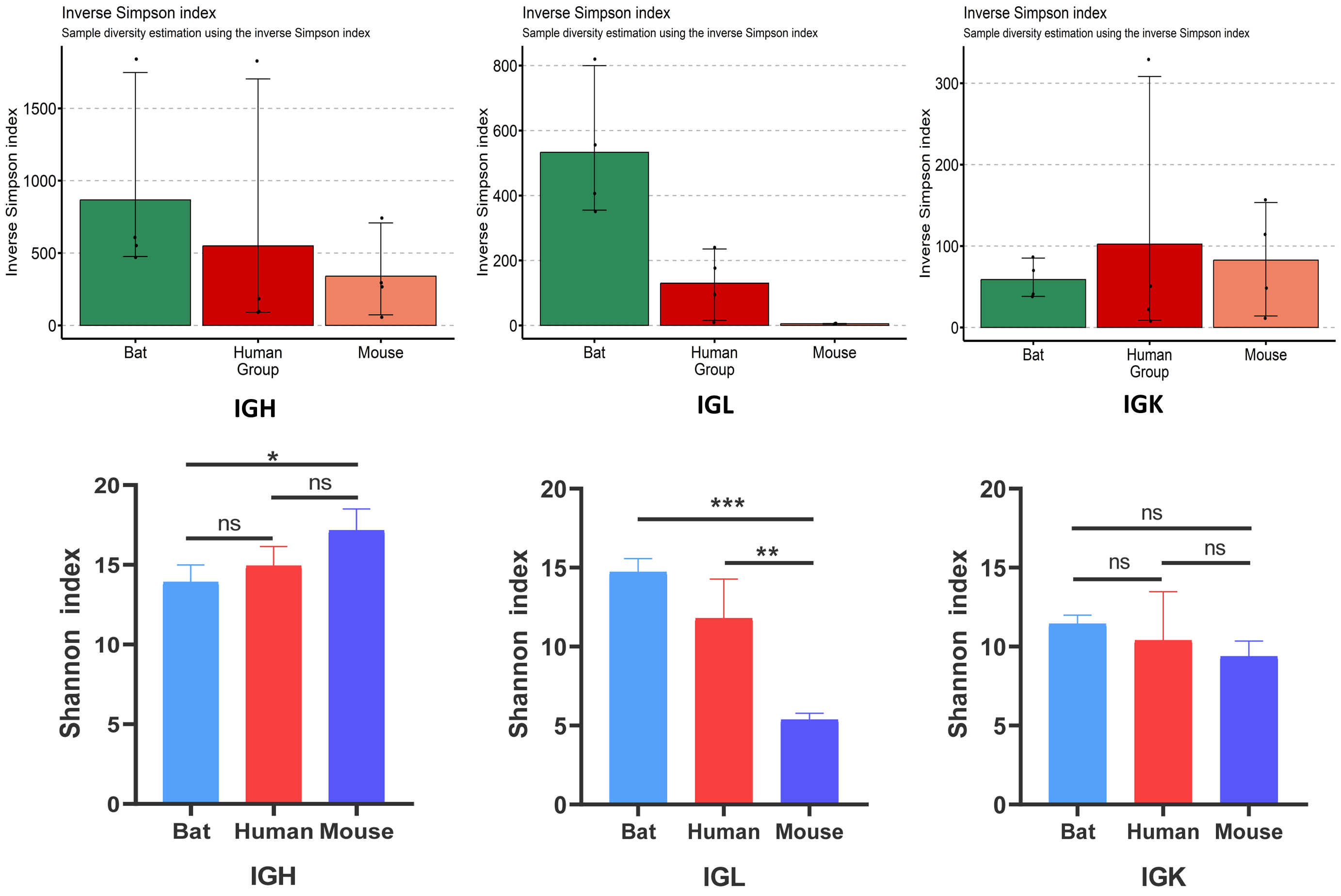
**

**Sup Fig 14.** Bat, human, and mouse IGH/IGL/IGKomic library diversity（Kruskal-Wallis H Test; one-way analysis of variance, P <0.05）

**
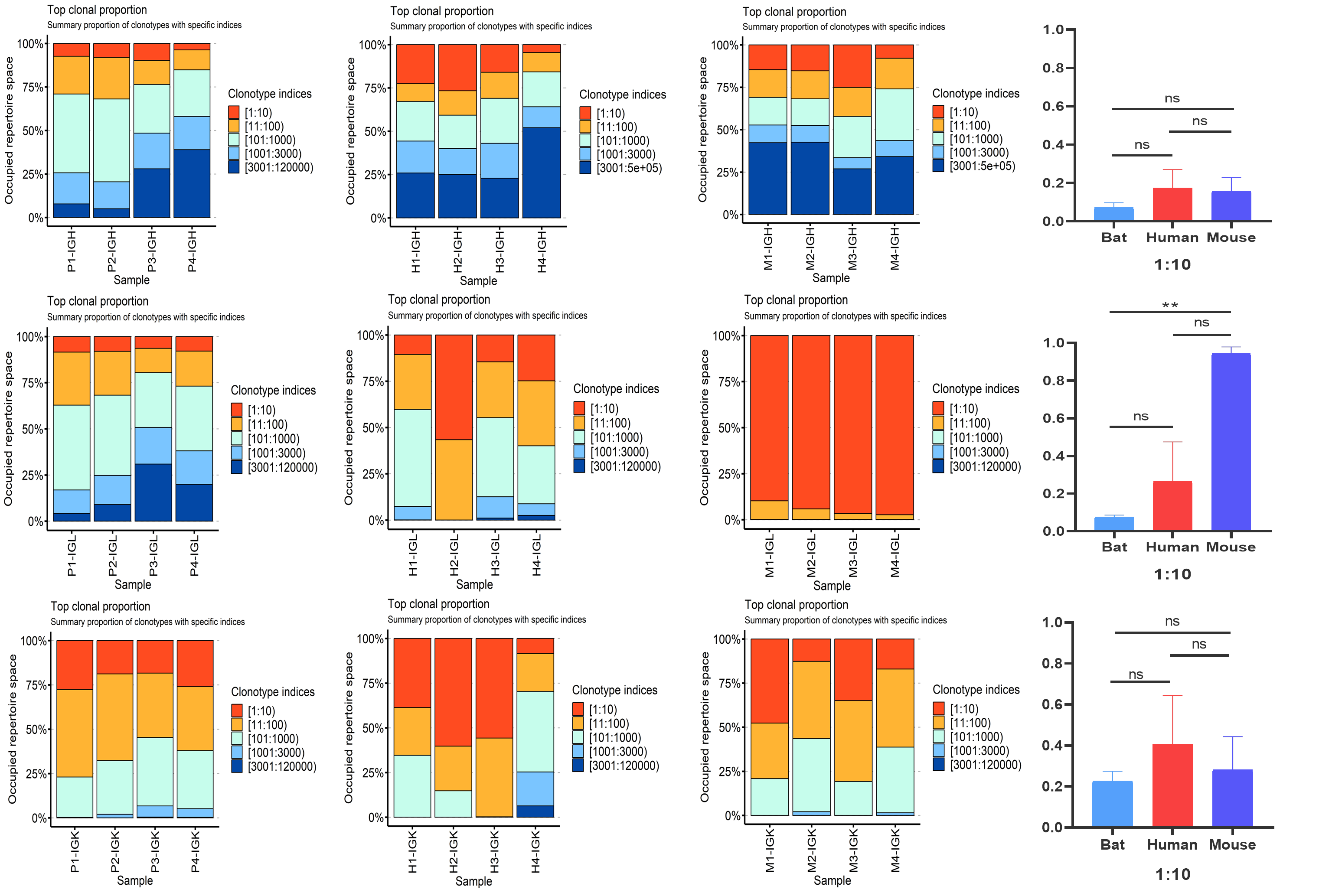
**

**Sup Fig 15.** Frequency distribution of IGH/IGL/IGK high-frequency clones in bats, humans, and mice（Kruskal-Wallis H Test,P <0.05）

**
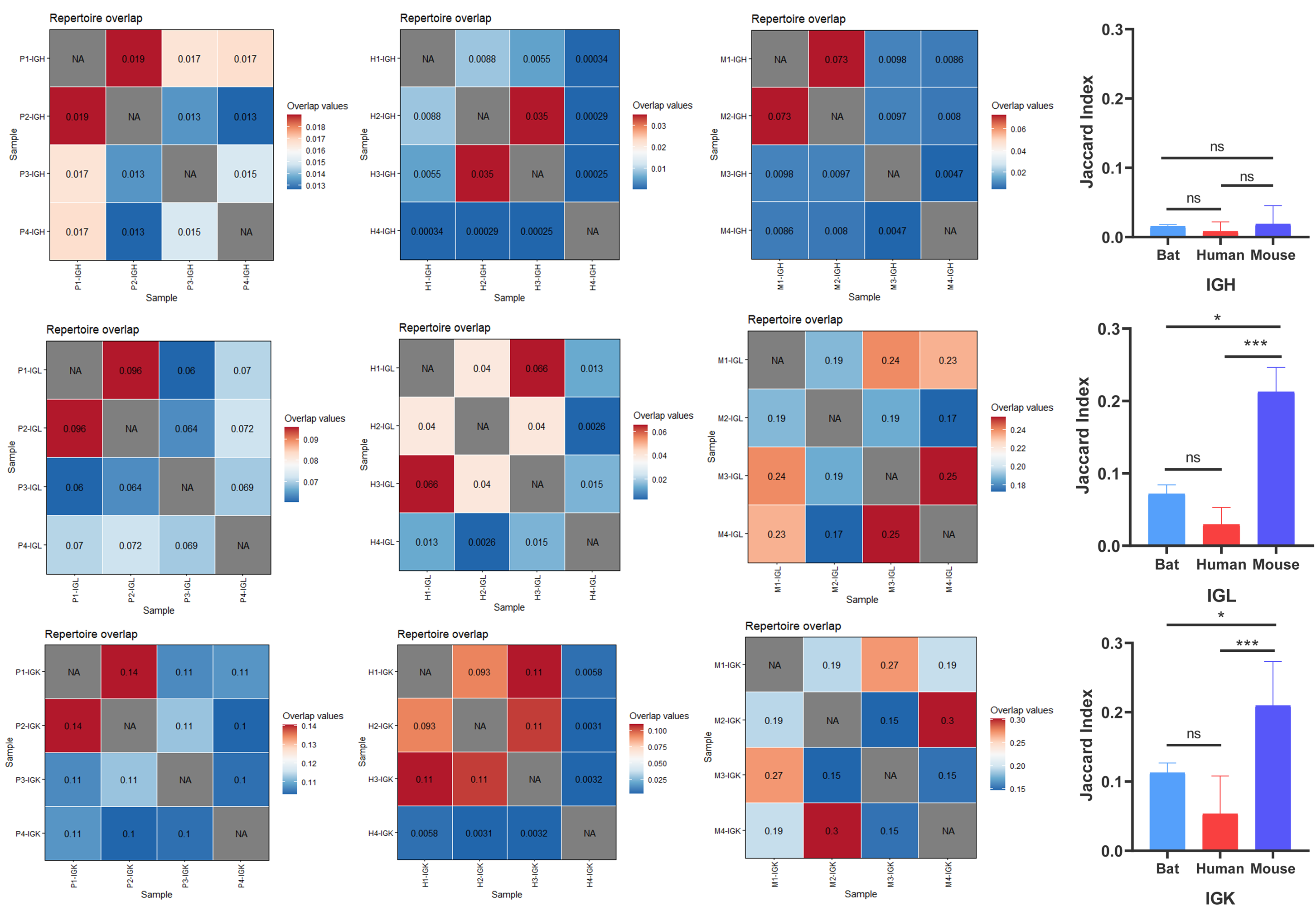
**

**Sup Fig 16.** Analysis of the IGH/IGL/IGK CDR3 overlap index in bats, humans, and mice（Kruskal-Wallis H Test,P <0.05）
